# Sleep trajectories across three cognitive‐aging pathways in community older adults

**DOI:** 10.1002/alz.70159

**Published:** 2025-05-02

**Authors:** Afsara B. Zaheed, Amanda L. Tapia, Nina Oryshkewych, Bradley J. Wheeler, Meryl A. Butters, Daniel J. Buysse, Yue Leng, Lisa L. Barnes, Andrew Lim, Lan Yu, Adriane M. Soehner, Meredith L. Wallace

**Affiliations:** ^1^ Department of Psychiatry University of Pittsburgh Pittsburgh Pennsylvania USA; ^2^ Department of Quantitative Health Sciences Mayo Clinic Rochester Minnesota USA; ^3^ School of Computing and Information University of Pittsburgh Pittsburgh Pennsylvania USA; ^4^ Clinical and Translational Science Institute University of Pittsburgh Pittsburgh Pennsylvania USA; ^5^ Department of Psychiatry and Behavioral Sciences University of California San Francisco San Francisco California USA; ^6^ Department of Neurological Sciences and Rush Alzheimer's Disease Center Rush University Medical Center Chicago Illinois USA; ^7^ Department of Neurology University of Toronto Ottawa Ontario Canada; ^8^ Department of Medicine University of Pittsburgh Pittsburgh Pennsylvania USA; ^9^ Department of Statistics University of Pittsburgh Pittsburgh Pennsylvania USA

**Keywords:** actigraphy, dementia, mild cognitive impairment, rest–activity rhythms, sleep health, smoothing splines

## Abstract

**INTRODUCTION:**

Comparing sleep and rest–activity rhythms across different cognitive aging pathways can identify novel risk factors and potential mechanisms. However, our current understanding is restricted by differences in sleep measurement, limited longitudinal data, and heterogeneous cognitive aging processes.

**METHODS:**

We applied cubic splines to longitudinal self‐reported sleep and actigraphy data from 1449 participants in the Rush Memory and Aging Project and quantified differences in the levels and trajectories of sleep amount, regularity, and timing within and between three cognitive aging pathways: normal, stable mild cognitive impairment, dementia.

**RESULTS:**

Sleep amount was lowest in the dementia pathway prior to cognitive impairment but increased with age, most rapidly after dementia. Regularity declined across all pathways, most rapidly after cognitive diagnoses. Timing advanced across all pathways.

**DISCUSSION:**

Shorter sleep amount in cognitively healthy older adults may be a risk factor or prodromal indicator of dementia, while longer sleep amounts and decreasing regularity may reflect neurodegeneration.

**Highlights:**

- We quantified longitudinal changes in sleep across three cognitive‐aging pathways.
- We incorporated both subjective and objective measures of sleep health.
- Self‐report duration increased noticeably from before to after cognitive diagnosis.
- Sleep irregularity increased most prominently after cognitive diagnosis.
- Advances in sleep timing occurred in both normal and pathological aging.

## 1 BACKGROUND

Alterations in sleep and circadian rest–activity rhythms (RARs) are clinical features of Alzheimer's disease and related dementias (ADRD).^1^ Individuals with dementia show substantial increases in sleep variability and shifts toward earlier sleep timing compared to cognitively healthy adults.^2^ There is also growing evidence that some of these changes occur at preclinical stages,^3^, ^4^ suggesting potential bidirectional relationships between sleep/RARs and neurodegenerative processes.^5^, ^6^ Although some findings indicate that poor sleep^7^ and weakened RARs^8^, ^9^ are associated with higher risk of mild cognitive impairment (MCI) or dementia, these findings have not been consistent across studies.^10^ Thus, the changes in sleep and RARs that occur during healthy aging^11^ are not well delineated from those that are risk factors for—or consequences of—neurodegenerative disease.^12^ Clarifying the role of sleep and RARs in cognitive aging is crucial for the development of dementia screening tools and interventions to prevent or delay late‐life cognitive impairment and dementia.

One reason for the mixed evidence surrounding the role of sleep and RARs in cognitive health may be the multitude of ways that these features can be characterized, leading to inconsistencies across studies. The “multidimensional sleep health” framework^13^ provides a novel vantagepoint for resolving this challenge: it recognizes sleep health as a biobehavioral processes “adapted to individual, social, and environmental demands”;^13^ calls for a consideration of multiple dimensions of sleep and RARs (e.g., the amount, regularity, and timing); and acknowledges that these dimensions can be measured using various modalities, including actigraphy and self‐report.^14^, ^15^ Objective and subjective indicators capture distinct aspects of sleep–wake behaviors^16^, ^17^, ^18^ and may exert effects on health through different mechanisms.^19^ Moreover, although sleep and RARs are inherently related, sleep is characterized by physiological alterations that can influence brain health independent of circadian processes;^20^, ^21^ thus, both sleep and RARs may be informative for differentiating normal versus neurodegenerative cognitive aging. Discrepancies among self‐reported sleep, actigraphy sleep, and actigraphy RARs^22^ likely contribute to inconsistent findings regarding the role of sleep health in cognitive aging.

Aside from challenges related to sleep/RAR measurement, several other gaps in the literature may contribute to a lack of clarity in the role of sleep/RARs in cognitive aging. Most studies examine sleep and RARs at a single time point;^8^, ^23^, ^24^ however, longitudinal sleep measurements are needed to differentiate normal variations in aging versus existing neurodegeneration and preclinical dementia. Although some prior studies have incorporated longitudinal data, the methods used assumed immediate changes in RARs at diagnostic benchmarks such as dementia diagnosis.^9^ Gradual changes may better represent sleep health trajectories across different cognitive aging pathways; however, such modeling approaches require relatively sophisticated statistical methods. Last, individuals with MCI are often combined with those with dementia, but this heterogeneity may obfuscate observed associations between sleep health and dementia. While individuals with MCI are 3 to 5 times more likely to progress to dementia within 5 years,^25^, ^26^ etiologies other than neurodegenerative diseases may contribute to MCI, such as vascular disorders,^27^ resulting in a stable lower level of cognitive functioning or returns to normal cognition over time.^28^, ^29^ Thus, sleep health trajectories for individuals with stable MCI may differ from those who progress to dementia.

To address these limitations, we used flexible cubic spline models to estimate trajectories of sleep and RARs across three cognitive‐aging pathways: normal cognitive aging (“normal”), progression to stable MCI (“stable MCI”), and progression to dementia (“dementia”). Model estimates were used to address our primary aims: (1) to determine which sleep/RAR features exhibit meaningful changes within each cognitive pathway and (2) to determine which sleep/RAR features differ meaningfully between cognitive aging pathways prior to the onset of any cognitive diagnosis, and after the onset of MCI and/or dementia. We hypothesized that the normal trajectory would be characterized by a shift toward shorter, less regular, and earlier sleep, and that the MCI and dementia trajectories would show a larger magnitude in each of these changes.

## 2 METHODS

### 2.1 Participants and study procedures

Participants were from the Rush Memory and Aging Project (MAP), an ongoing longitudinal cohort study of adults aged 65+ without known dementia at enrollment.^30^ Beginning in 1997, MAP recruited participants from retirement communities and subsidized senior housing facilities throughout Chicagoland and northeastern Illinois in the United States. Annual visits include a clinical evaluation of cognitive status (i.e., no cognitive impairment, MCI, and ADRD) determined using a three‐stage process including computer scoring of cognitive tests, clinical judgment by a neuropsychologist, and diagnostic classification by a clinician (see details described elsewhere^31^). Between 2005 and 2018, participants also wore an Actical (Phillips Respironics) accelerometer on their non‐dominant wrist every ≈ 1 to 2 years for up to 10 days (median of 9 days), allowing for quantification of RARs and objective sleep features. Beginning in 2012, annual assessments incorporated a comprehensive 32‐item questionnaire of sleep health and sleep disorder symptoms, with specific items drawn from validated measures including the Pittsburgh Sleep Quality Index, Berlin Questionnaire, and Mayo Sleep Questionnaire.^32^

Consistent with our prior work with MAP,^33^ our analytic sample included participants aged ≥ 65, thereby emphasizing individuals with the greatest risk for MCI or dementia. We further refined the cohort by focusing only on Black and White participants, as participants from other racial categories comprised <1% of the full cohort. Because valid self‐report sleep, actigraphy sleep, and actigraphy RAR features were available for slightly different sets of participants at each visit, we developed a self‐reported sleep sample (*N* = 989, with 3243 longitudinal self‐reported sleep observations), an actigraphy sleep sample (*N* = 1223, with 4862 longitudinal actigraphy sleep observations), and an actigraphy RAR sample (*N* = 1196, with 4452 longitudinal RAR observations). There are *N* = 1449 unique participants across samples. Figure S1 in supporting information includes a flow chart detailing the derivations of the analytic samples.

The grant facilitating secondary data analysis of MAP was declared exempt by the University of Pittsburgh Institutional Review Board (PRO17050218). All original data collection was performed in accordance with the ethical standards established in the 1964 Declaration of Helsinki and its later amendments, and all participants provided written informed consent to participate in longitudinal studies of sleep and health. In this secondary data analysis, we addressed diversity, equity, and inclusivity (DEI) through the lens of a socioecological framework^34^; this led to careful statistical adjustment for race, ethnicity, and sex/gender, as well as potential physical and social determinants that may be upstream causes in observed racial/ethnic or sex/gender inequities in sleep and cognition (see section 2.5).

**RESEARCH IN CONTEXT**

1. **Systematic review**: The authors reviewed the literature using PubMed and Google Scholar. Studies indicate that different aspects of sleep health change after dementia, but evidence is mixed regarding the role of sleep health prior to cognitive diagnosis. Changes in sleep health that occur during healthy aging, stable mild cognitive impairment, and dementia are not well delineated.
2. **Interpretation**: Prior mixed findings may stem from studies inconsistently considering actigraphy sleep, actigraphy rest–activity rhythms, and self‐reported sleep, with most studies focusing on features measured at a single time point. An examination of longitudinal changes in multimodal sleep health across different pathways of cognitive aging is needed.
3. **Future directions**: Within‐person changes in the amount and regularity of sleep health emerged as potential risk factors for future dementia. Future studies should evaluate the underlying mechanisms and assess whether modifying these trajectories can slow and/or delay pathological cognitive aging.

### 2.2 Actical data processing

Actical is a waterproof wristwatch‐like accelerometer with acceleration primarily measured in an axis parallel to the device face with some sensitivity to movements in other axes. Activity counts are integrated for each 15 second period, or “epoch.” Consistent with prior work,^35^ we flagged periods with > 4 consecutive hours of no movement (representing Actical removal) and excluded the entire 24 hour period surrounding this from subsequent analysis. With these data, we estimated both parametric and non‐parametric RARs.

For parametric RARs, we used extended cosine models to extract features describing the shape of the estimated curve for each participant,^36^ requiring at least partial model convergence to use the estimated parameters. This was performed using the RAR package in R.^37^ For non‐parametric RARs, we quantified patterns of rest and activity not constrained by parametric model shapes using the nparACT package in R.^38^ We required ≥ 5 days of valid Actical recording for the session to be considered in the analytic RAR sample.

To obtain actigraphy estimates of sleep, we applied the Crespo algorithm^39^ using pyActigraphy in Python.^40^ This approach accommodated the raw Actical activity data and has been validated against other gold‐standard algorithms. Because the Crespo algorithm was validated based on 60 second epochs, we down‐sampled the 15 second epoch Actical data to 60 second epochs using a finite impulse response filter with Hamming window prior to using the algorithm. We set a maximum duration of 18 hours to exclude potentially invalid sleep intervals. The Crespo algorithm identifies the timing of sleep onset and offset, and from these the sleep interval length can also be computed. It does not indicate periods of awakening between rest onset and offset. We required ≥ 5 days of both valid Actical recording and observed sleep bouts for the session to be included in the analytic sleep sample.

### 2.3 Sleep health outcomes

Although the classic sleep health framework considers up to six sleep dimensions,^13^ the three dimensions that are directly modifiable through behavioral interventions^41^ are the “ART” of sleep: amount (duration of the main sleep or rest interval), regularity (consistency of sleep timing or rhythms), and timing (midpoint of sleep or active interval). Informed by prior work examining the dimensionality of sleep health in older adults,^42^ we selected self‐reported sleep and actigraphy‐based sleep and RAR measures to represent each of the ART domains. Definitions of each feature are provided in Table 1.

**TABLE 1:** Sleep and rest–activity rhythm outcome measures.

Measurement type	Sleep health domain		
	Amount	Regularity	Timing
Self‐reported sleep	*Total Sleep Time* (TST; hours and minutes): the typical duration of nighttime sleep over the past month. Higher values indicate longer sleep duration.	—	*Midpoint* (military clock time): the midpoint between self‐reported bedtime and wake time over the past month.
Actigraphy sleep	*Rest Interval Length* (hours and minutes): the average interval of the rest period across days per annual visit. Higher values indicate longer a longer rest interval length.	*Midpoint Standard Deviation*: the log‐transformed standard deviation of the midpoint across days. Higher values indicate more variability in sleep timing.	*Average Midpoint* (military clock time): the midpoint of the rest interval.
Actigraphy rest–activity rhythms	*Alpha*: the time spent in periods of activity (peaks) relative to rest (troughs). Higher values indicate more time spent in a rest state. Valid range: [−1, 1]	*Interdaily Stability* (IS): the degree of consistency of rest‐activity patterns from one day to the next. Higher values indicate greater stability across days. Valid range: [0, 1]. *Intradaily Variability* (IV): the degree of fragmentation of rest–activity rhythms within a 24 hour period. Higher values indicate greater variability within a day. Valid range: [0, 2].	*Acrophase* (military clock time): the timing of peak activity within a 24 hour period.

#### 2.3.1 Amount

For RARs, we selected “alpha” from the extended cosine model to measure the relative duration of nighttime rest or inactivity to daytime activity.^42^ For actigraphy sleep, we selected the average “rest interval length.” For self‐reported sleep, we selected the item “During the past month, how many hours/minutes do you usually sleep at night?” as a measure of sleep duration.

#### 2.3.2 Regularity

For RARs, we selected non‐parametric “intradaily variability” (IV) and “interdaily stability” (IS). These features index the variability and stability of RARs within and across days, respectively.^9^ For actigraphy sleep, we selected the standard deviation of the rest interval midpoint (“actigraphy midpoint variability”). No self‐reported measures of sleep regularity were available in MAP.

#### 2.3.3 Timing

For RARs, we selected “acrophase” from the extended cosine model; it represents the time of peak activity.^42^ For actigraphy sleep, we selected the average of the rest interval midpoint (“actigraphy midpoint”) to represent sleep timing.^42^ For self‐reported sleep, we computed the midpoint of self‐reported bed and wake‐up times (self‐reported midpoint). Bed time and wake time were obtained by asking, “During the past month, when have you usually gone to bed at night/gotten up in the morning?”

### 2.4 Cognitive pathways

Because of the nature of follow‐up in the MAP study, some participants had cognitive diagnoses either prior to their first sleep/RAR visit or after their final sleep/RAR visit. The totality of cognitive diagnosis information from before, during, and after sleep/RAR data collection was used to group participants into one of three pathways describing their cognitive trajectory in the MAP study.

1. Normal cognitive aging (“normal”): Throughout their time in the MAP study, participants had no dementia diagnosis and never had two consecutive years of MCI.
2. Progression to stable MCI (“stable MCI”): At any point during their time in the MAP study—including before or after the first or last sleep/RAR observation—participants had two consecutive MCI diagnoses. Given that a person with a MCI diagnosis reverts to normal cognitive status ≈ 30% to 40% of the time,^28^, ^29^, ^43^ two consecutive diagnoses serve to reduce heterogeneity in the MCI group and index stable MCI. Participants on this pathway never received a dementia diagnosis.
3. Progression to dementia (“dementia”). At any point during their time in the MAP study—including before or after the first or last sleep/RAR observation—participants had a dementia diagnosis. Participants could also receive MCI diagnoses prior to dementia diagnosis, but this was not required for inclusion in this pathway.

### 2.5 Covariates

We selected covariates that were potentially sleep related, that could confound associations among indices of sleep health and cognitive status, and that may represent upstream causes in observed racial/ethnic inequities in sleep and cognition. Covariates measured at either the initial study baseline or the first analytic sample visit were self‐reported sex (male/female), racial identity (Black/African American or White), Hispanic ethnic identity (yes/no), years of education, marital status (never married, married, widowed, or divorced/separated), average grams of alcohol consumed per day during the past 12 months, and early life socioeconomic status (*Z* score composite of maternal and paternal education and number of household children). Covariates measured at each visit included number of self‐reported medical conditions (range: 0–6), use of insomnia medication (yes/no), depressive symptoms (range: 0–9; higher scores indicate greater depressive symptoms), instrumental activities of daily living (range: 0–8; higher scores indicate greater disability), the percentage of valid days with sleep detected by the Crespo algorithm (actigraphy sleep models only), an indicator variable for participant death (to account for incomplete follow‐up resulting from death vs. dropout for other reasons), and global cognitive function. Global cognitive function is a composite *Z* score calculated from a battery of 19 cognitive tests assessing episodic memory, semantic memory, working memory, visuospatial ability, and perceptual speed;^30^ positive scores indicate above‐average global cognition. This measure was included because participants within a particular pathway may have varying levels of cognition, which may be related to sleep and RARs. Additional covariate details are documented elsewhere.^33^, ^44^

### 2.6 Statistical analysis

#### 2.6.1 Preliminary analyses

We summarized and compared distributions of missing data, covariates, cognitive status, and years of follow‐up within each cognitive pathway and in the overall sample. Because the self‐reported sleep, actigraphy sleep, and RAR samples included slightly different participants and follow‐up time points, we compared these characteristics across samples using descriptive statistics.

#### 2.6.2 Cubic spline models

We used linear mixed effects cubic smoothing splines^45^, ^46^ to estimate longitudinal sleep/RAR trajectories of each of the three cognitive pathways simultaneously. Age (vs. year in study) was selected as the time marker because of biological changes in sleep/RARs that occur even with healthy aging^14^, ^33^ and the wide age range at the initial sleep/RAR visit (ages 65–100). Models for actigraphy sleep and RARs were weighted by the number of valid days of actigraphy.

Participants could contribute data to only one of the three cognitive pathways across follow‐up (normal, stable MCI, or dementia). These pathways were allowed to differ in their average levels and their trajectories through main effect pathway terms and age‐by‐pathway interaction terms. Alterations in sleep and RAR trajectories surrounding a cognitive diagnosis were modeled using cubic spline “knots,” which allowed for sleep/RAR changes to occur gradually and non‐linearly (in contrast to the sharp “elbow” seen in linear spline models). Specifically, the stable MCI path included a spline knot at the age of MCI onset, allowing for the sleep/RAR trajectory to be further altered at MCI onset. The dementia path included spline knots at two possible locations: the age of stable MCI onset (if a stable MCI diagnosis was recorded prior to dementia) and the age of dementia onset. By incorporating “knots” at MCI and/or dementia, the pathways were divided into cognitive aging segments. Specifically, in the stable MCI pathway, participants could contribute data to an initial period of normal cognition and/or a subsequent period of MCI. On the dementia pathway, participants could contribute data to an initial period of normal cognition, intermediary MCI, and/or a final period of dementia. See Figures S2 and S3 in supporting information for additional details.

To follow best‐practice guidelines on fitting cubic spline models^45^, ^46^ and allow for gradual changes across age and cognitive diagnoses, we specified each initial model to include age, squared age, cubic age, cognitive path, interactions between all age terms with cognitive paths, cubic splines at MCI onset age, a cubic spline at dementia onset age, and all covariates. To build a more parsimonious model, higher order terms for age interactions, main effects of age, and splines were excluded from each model one at a time based on the highest *p* value, if not statistically significant (*α* = 0.05). We included random subject and age effects to account for within‐subject correlations in all models. All models used the *lme* function from the *nlme* package.^47^

#### 2.6.3 Contrasts

The fitted spline models were used to estimate the sleep or RAR outcome for a typical MAP participant (i.e., using average covariate levels) for a specified pathway, age, and cognitive diagnosis. Leveraging these estimates, we tested within‐ and between‐pathway differences in sleep/RAR levels using standardized contrasts (denoted *d*) and their 95% confidence intervals (CIs) at specified benchmark ages selected based on our analytic sample. Beginning with the median age of the first sleep/RAR visit (age 82), we considered four equally spaced benchmark ages that allowed for at least 4 years within each potential segment of a pathway: 82, 86, 90, and 94. For the normal pathway, we assumed participants had normal cognition between ages 82 and 94. For the stable MCI pathway, we assumed participants had normal cognition from ages 82 to 86 and MCI from ages 86.1 to 94. For the dementia pathway, we assumed participants had normal cognition from ages 82 to 86, MCI from 86.1 to 90, and dementia from ages 90.1 to 94.

In addition to this primary set of benchmark ages, we also considered four secondary sets of benchmark ages designed to evaluate the robustness of our findings. (1) We shifted the original set of benchmark ages 2 years earlier {80, 84, 88, 92}. (2) We shifted the original benchmark ages 2 years later {84, 88, 92, 96}. (3) We used 3‐year (instead of 4‐year) intervals {82, 85, 88, 91}. (4) We allowed an earlier/longer period of normal cognition {80, 86, 90, 94}, which was of was of interest because of its potential relevance for screening. We chose not to examine scenarios with longer periods of MCI or dementia because there are relatively few participants with periods of MCI and/or dementia longer than 4 years in the MAP study.

To determine which sleep/RAR features exhibit meaningful within‐pathway changes (Aim 1), we used the estimated spline models to compute overall standardized mean change (*d* [95% CI]) in sleep/RAR levels from the initial benchmark age to the final benchmark age. We used standard practices for interpreting effect size, with |*d *| < 0.2 indicating very small effects, |*d*| = 0.2 to 0.49 indicating small effects, |*d*| = 0.5 to 0.79 indicating moderate effects, and |*d*| ≥ 0.8 indicating large effects.^48^ For features with moderate‐to‐large overall changes (|*d*| ≥ 0.50) in our primary set of benchmark ages, we explored changes during key age segments of interest within each pathway (ages 82–86, 86.1–90, and 90.1–94). To determine which sleep/RAR features exhibit meaningful differences between cognitive pathways prior to the onset of any cognitive diagnosis (Aim 2a) and after potential cognitive diagnosis (Aim 2b), we used the estimated spline models to compute the standardized mean difference (*d* [95% CI]) between the sleep/RAR levels for each pair of pathways at our first benchmark age (assuming normal cognition in all three pathways) and our last benchmark age (assuming normal cognition for the normal pathway; MCI diagnosis at the second benchmark age for the stable MCI and dementia pathways; and dementia diagnosis at the third benchmark age for the dementia pathway).

Across analyses, our primary focus was on results that are both clinically meaningful, as indicated by at least a moderate effect size (|*d*| ≥ 0.50), and statistically significant after multiple comparison correction based on the Benjamini–Hochberg family‐wise error approach.^49^ Specifically, for each aim, we adjusted for multiple comparisons within each domain (amount, regularity, duration). However, at times we also discuss results that are non‐significant or with smaller effect sizes to highlight broader trends across the features. Contrasts were estimated using the contrast and emmeans functions from the emmeans package.^50^

## 3 RESULTS

Descriptive characteristics of the full analytic sample (*N* = 1449) and each cognitive pathway are summarized in Table 2. Briefly, participants’ first sleep/RAR observation occurred at a median age of 82 (range 65–100) and participants had a mean (standard deviation [SD]) of 5.03 (4.78) total years of cognitive diagnosis data (range 1–15). Descriptive statistics of each separate analytic sample are provided in Table S1 in supporting information. The three analytic samples (self‐reported sleep, actigraphy sleep, actigraphy RAR) had comparable sociodemographic characteristics. However, the self‐report sleep sample had a somewhat greater percentage of participants in the normal pathway (66% the self‐report sample; 55%–56% in the actigraphy samples) and a lower proportion of participants who died during follow‐up (35% in the self‐report sample; 55% in the actigraphy samples).

**TABLE 2:** Participant characteristics from the initial study visit. The sample summarized includes all participants in the self‐report sleep sample, actigraphy sleep sample, and/or the actigraphy rest–activity rhythm sample.

	Normal pathway (*N* = 855)	Stable MCI pathway (*N* = 194)	Dementia pathway (*N* = 400)	All (*N* = 1449)
**Sociodemographic characteristics**				
Age at initial visit, mean (SD)	79.4 (7.23)	83.4 (5.89)	83.9 (6.26)	81.2 (7.12)
Race, Black, *N* (%)	36 (4.2%)	18 (9.3%)	22 (5.5%)	76 (5.2%)
Sex, Female, *N* (%)	670 (78.4%)	128 (66.0%)	292 (73.0%)	1090 (75.2%)
Years of education, mean (SD)	15.5 (3.01)	14.9 (2.95)	14.9 (3.14)	15.3 (3.05)
Not Spanish/Hispanic/Latin origin, *N* (%)	837 (97.9%)	192 (99.0%)	392 (98.0%)	1421 (98.1%)
Marital status, *N* (%)				
Never married	59 (6.9%)	13 (6.7%)	22 (5.5%)	94 (6.5%)
Divorced/Separated	104 (12.2%)	28 (14.4%)	35 (8.8%)	167 (11.5%)
Married	373(43.6%)	64 (33.0%)	143 (35.8%)	580 (40.0%)
Widowed	319 (37.3%)	89 (45.9%)	200 (50.0%)	608 (42.0%)
Early life SES, mean (SD)	0.152(0.704)	−0.0561 (0.708)	−0.0463 (0.683)	0.0695 (0.705)
***Health characteristics***				
Died during follow‐up, *N* (%)	304 (35.6%)	112 (57.7%)	301 (75.3%)	717 (49.5%)
Global cognitive function,^a^ mean (SD)	0.354 (0.449)	−0.145 (0.472)	−0.459 (0.766)	0.0631 (0.665)
Medical conditions,^b^ mean (SD)	1.49 (1.07)	1.57 (1.01)	1.50 (1.09)	1.50 (1.07)
IADLs,^c^ mean (SD)	0.749 (1.29)	1.10 (1.62)	1.64 (2.10)	1.04 (1.64)
Insomnia medications, *N* (%)	79 (9.2%)	12 (6.2%)	35 (8.8%)	126 (8.7%)
CESD (sleep item removed),^d^ mean (SD)	0.799 (1.27)	0.804 (1.24)	1.16 (1.57)	0.900 (1.37)
Alcohol per day (grams), mean (SD)	6.10 (14.7)	5.36 (12.5)	4.77 (10.7)	5.63 (13.4)
Age at 2 consecutive MCI dx,^e^ mean (SD)	NA	85.8 (6.28)	85.6 (5.84)	85.7 (6.05)
Age at first dementia dx, mean (SD)	NA	NA	88.2 (6.70)	88.2 (6.70)
***Study characteristics***				
Years of follow‐up after initial visit,^f^ mean (SD)	3.93 (3.88)	4.19 (3.67)	4.15 (3.59)	4.03 (3.78)
Number of repeated sleep/RAR measures,^g^ mean (SD)	4.21 (3.24)	4.39 (3.10)	4.16 (2.94)	4.22 (3.14)

Abbreviations: CESD, Center for Epidemiological Studies‐Depression; Dx, diagnosis; IADLs, Instrumental Activities of Daily Living; MCI, mild cognitive impairment; RAR, rest–activity rhythm; SD, standard deviation; SES, socio‐economic status.

^a^ Measured using an averaged *Z* score composite index based on paternal and maternal years of education and number of children in the household during childhood.

^b^ Valid range 0–6, considering clinician diagnosis of stroke or self‐reported hypertension, diabetes, heart disease, cancer, or thyroid disease.

^c^ Measure adapted from the Duke Older Americans Resources and Services project with higher scores indicating greater disability.

^d^ Measured using 9‐item version of the CES‐D with the sleep item removed to avoid tautology.

^e^ Missing *N* = 196 (49%) participants in the dementia path who did not have an MCI diagnosis, and 1051 (72.5%) participants in the overall sample who did not have an MCI diagnosis.

^f^ Computed as the age at the last visit with any sleep/RAR data minus the age at the first visit with any sleep/RAR data. This represents the years elapsed between the initial and last visit with sleep/RAR data.

^g^ The total number of observed sleep and/or RAR measures.

**FIGURE 1:**
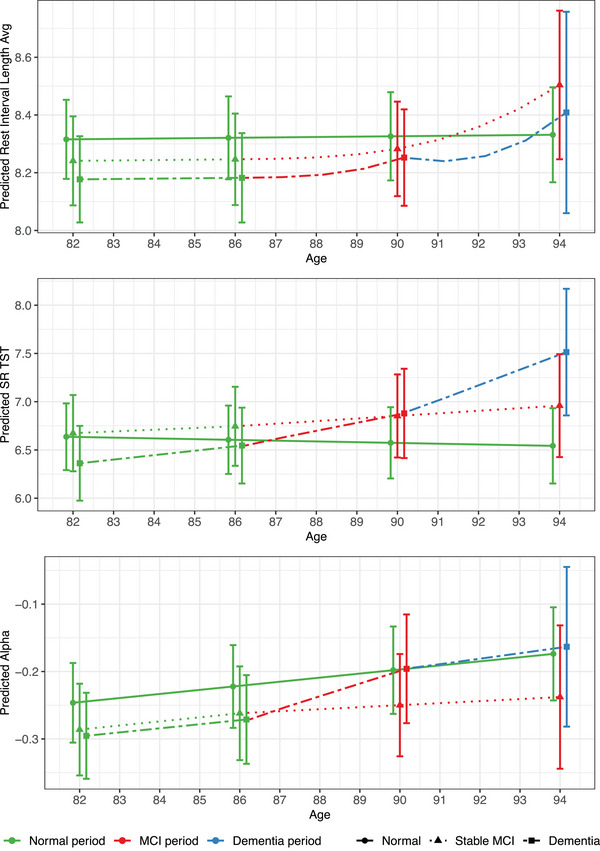
Trajectories for sleep amount outcomes. Unstandardized sleep amount trajectories are depicted for a “typical” participant from the analytic study sample who either remained cognitively unimpaired between ages 82 and 94 (normal pathway), transitioned from normal cognition to MCI at age 86 without subsequent dementia (stable MCI pathway), or transitioned to MCI at age 86 and subsequently to dementia at age 90 (dementia pathway). MCI, mild cognitive impairment; SR, self‐report; TST, total sleep time.

**FIGURE 2:**
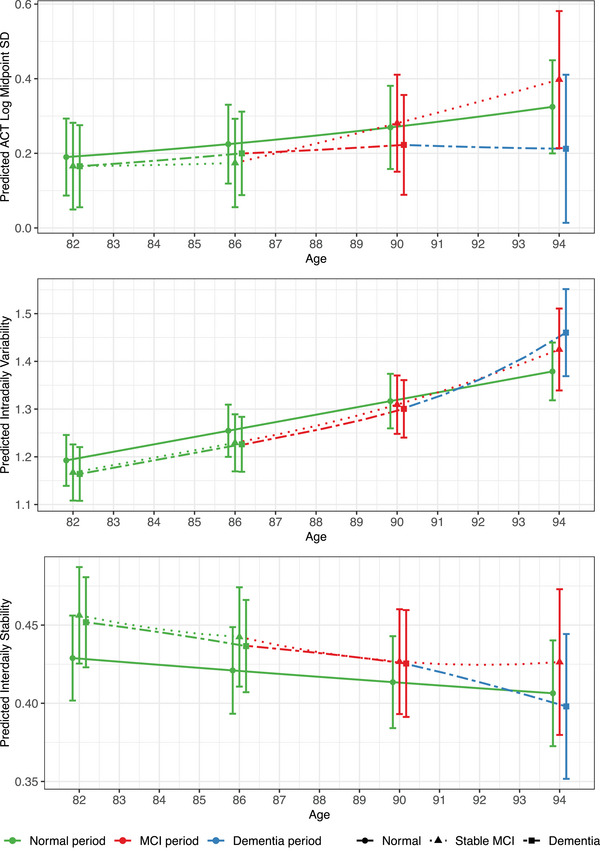
Estimated trajectories for sleep regularity outcomes. Unstandardized sleep regularity trajectories are depicted for a “typical” participant from the analytic study sample who either remained cognitively unimpaired between ages 82 and 94 (normal pathway), transitioned from normal cognition to MCI at age 86 without subsequent dementia (stable MCI pathway), or transitioned to MCI at age 86 and subsequently to dementia at age 90 (dementia pathway). ACT, actigraphy; MCI, mild cognitive impairment; SD, standard deviation.

**FIGURE 3:**
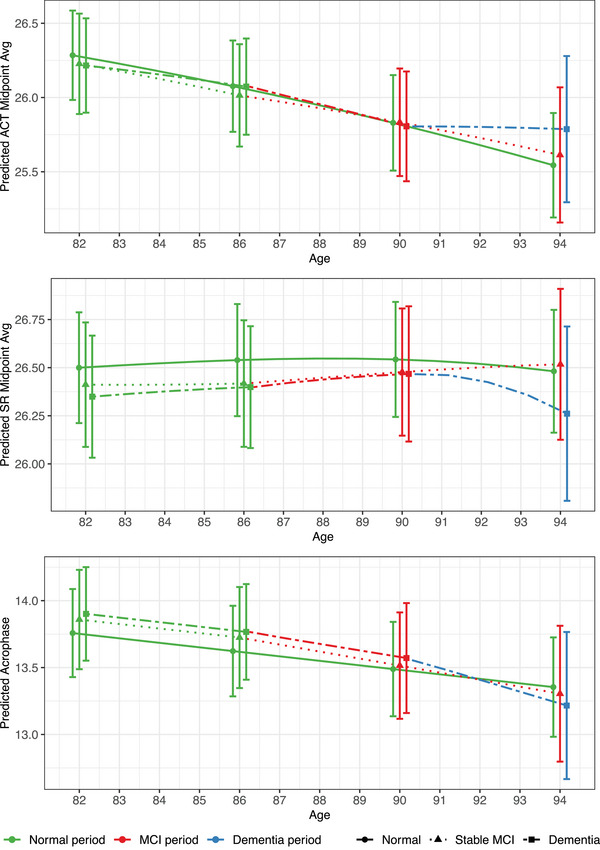
Estimated trajectories for sleep timing outcomes. Unstandardized sleep timing trajectories are depicted for a “typical” participant from the analytic study sample who either remained cognitively unimpaired between ages 82 and 94 (normal pathway), transitioned from normal cognition to MCI at age 86 without subsequent dementia (stable MCI pathway), or transitioned to MCI at age 86 and subsequently to dementia at age 90 (dementia pathway). ACT, actigraphy; MCI, mild cognitive impairment; SR, self‐report.

Estimated trajectories of unstandardized sleep and RAR outcomes for the amount, regularity, and timing of each pathway are depicted in Figures 1, 2, 3, respectively. These figures illustrate the non‐linear nature of the sleep and RAR trajectories with age, along with further trajectory alterations in the years surrounding MCI and/or dementia diagnoses. They also illustrate the differences in sleep and RARs between the three cognitive aging pathways and the changes within each cognitive pathway. The accompanying cubic spline model parameter estimates are provided in Tables S2–S4 in supporting information.

### 3.1 Longitudinal changes within each cognitive pathway

Table 3 provides standardized changes and 95% CIs from ages 82 to 94 for each cognitive aging pathway and each sleep/RAR outcome. Below, we describe the magnitude of the effect size (very small, small, moderate, or large) and report the unstandardized estimate (i.e., in its original scale) for each sleep/RAR outcome. A descriptive summary of within‐pathway changes along key age segments (82–86, 86.1–90, and 90.1–94) and the full follow‐up age range (82–94) is provided in Table 4, with full details in Table S5 in supporting information.

**TABLE 3:** Estimated standardized within‐pathway changes (*d* [95% CI]) in self‐report sleep, RAR, and actigraphy sleep features.

Feature	Normal path^a^	Stable MCI path^b^	Dementia path^c^	Group differences^d^
***Amount***				
Self‐reported total sleep time	−0.075 (–0.192, 0.042)	0.227 (–0.131, 0.584)	**0.917 (0.473, 1.360)**	Dementia > normal, MCI
Actigraphy rest interval length	0.013 (–0.041, 0.068)	**0.222 (0.034, 0.410)**	0.196 (–0.071, 0.462)	MCI > normal
Actigraphy alpha	**0.266 (0.172, 0.360)**	0.177 (–0.180, 0.533)	**0.484 (0.111, 0.857)**	
***Regularity***				
Actigraphy midpoint standard deviation	**0.229 (0.100, 0.357)**	**0.394 (0.091, 0.696)**	0.079 (–0.217, 0.376)	
Actigraphy interdaily stability	−0.205 (–0.372, –0.037)	−0.283 (–0.621, 0.056)	**−0.422 (−0.717, −0.127)**	
Actigraphy intradaily variability	**0.698 (0.613, 0.783)**	**0.965 (0.702, 1.228)**	**1.109 (0.840, 1.377)**	MCI, dementia > normal
***Timing***				
Self‐reported midpoint	−0.020 (−0.181, 0.142)	0.112 (−0.222, 0.446)	−0.093 (−0.474, 0.287)	
Actigraphy rest interval midpoint	**−0.534 (−0.671, −0.397)**	**−0.443 (−0.717, −0.168)**	**−0.309 (−0.593, −0.026)**	
Actigraphy acrophase	**−0.320 (−0.422, −0.218)**	**−0.439 (−0.767, −0.111)**	**−0.542 (−0.885, −0.200)**	

*Notes*: Bold findings are significant after Benjamini–Hochberg multiple comparison adjustment within each feature type (amount, regularity, timing). Estimates are standardized such that values with magnitudes of |*d*| < 0.20 are considered very small, |*d*| ≥ 0.20−0.49 are considered small, |*d*| ≥ 0.50−0.79 are considered moderate, and |d|≥0.80 are considered large.

Abbreviations: CI, confidence interval; MCI, mild cognitive impairment.

^a^ Estimates are for a “typical” participant on the normal pathway, with no cognitive diagnosis across follow‐up and average levels of covariates.

^b^ Estimates are for a “typical” participant on the stable MCI pathway, with MCI at age 86, no dementia diagnosis, and average levels of covariates.

^c^ Estimates are for a “typical” participant on the dementia pathway, with MCI at age 86, dementia at age 90, and average levels of covariates.

^d^ Statistically significant differences in sleep health trajectories between cognitive aging pathways.

**TABLE 4:** Descriptive summary of sleep/RAR trajectories across each cognitive aging pathway by study period.

Sleep/RAR feature	Pathway	Initial normal cognition period (ages 82–86)	MCI period (ages 86–90)	Dementia period (ages 90–94)	Full follow‐up period (ages 82–94)
**Amount**					
Self‐reported Total Sleep Time	Normal	–	–	–	–
	MCI	–	–	–	–
	Dementia	Very small ↑	Small ↑	**Moderate ↑**	**Large ↑**
Actigraphy rest interval length	Normal	–	–	–	–
	MCI	–	Very small ↑	Very small ↑	Small ↑
	Dementia	–	Very small ↑	–	–
Actigraphy alpha	Normal	Very small ↑	Very small ↑	Very small ↑	Small ↑
	MCI	Very small ↑	–	–	–
	Dementia	Very small ↑	Small ↑	–	Small ↑
**Regularity**					
Actigraphy midpoint variability	Normal	Very small ↑	Very small ↑	Very small ↑	Small ↑
	MCI	–	Very small ↑	Very small ↑	Small ↑
	Dementia	Very small ↑	–	–	–
Actigraphy interdaily stability	Normal	Very small ↓	Very small ↓	–	–
	MCI	Very small ↓	–	–	*–*
	Dementia	Very small ↓	–	–	Small ↓
Actigraphy intradaily variability	Normal	Small ↑	Small ↑	Small ↑	**Moderate ↑**
	MCI	Small ↑	Small ↑	Small ↑	**Large ↑**
	Dementia	Small ↑	Small ↑	**Moderate ↑**	**Large ↑**
**Timing**					
Self‐reported Midpoint	Normal	–	–	–	–
	MCI	–	–	–	–
	Dementia	–	–	–	–
Actigraphy rest interval midpoint	Normal	Very small ↓	Very small ↓	Small ↓	**Moderate ↓**
	MCI	Very small ↓	Very small ↓	Very small ↓	Small ↓
	Dementia	Very small ↓	Very small ↓	–	Small ↓
Actigraphy acrophase	Normal	Very small ↓	Very small ↓	Very small ↓	Small ↓
	MCI	Very small ↓	Very small ↓	Very small ↓	Small ↓
	Dementia	Very small ↓	–	Small ↓	**Moderate ↓**

*Notes*: The descriptors (very small, small, moderate, and large) correspond to effects that are statistically significant with mean standardized changes of |*d*| < 0.2, = 0.2−0.49, = 0.5−0.79, and ≥ 0.8, respectively. Bolded terms highlight the moderate and large effects. ↑ = increase or positive change over time; ↓ = decrease or negative change over time. Dashes (“–”) indicate the effect was not statistically significant after Benjamini–Hochberg correction.

Abbreviations: MCI, mild cognitive impairment; RAR, rest–activity rhythm.

#### 3.1.1 Amount

In the normal pathway, there was a statistically significant but small increase in actigraphy alpha (unstandardized value changing from −0.25 at age 82 to −0.17 at age 94) suggesting somewhat greater time in rest versus activity with older age. However, there were no changes in either self‐reported duration or actigraphy rest interval length for the normal pathway. In the stable MCI pathway, there was a statistically significant but small increase in actigraphy rest interval length (08:14 to 08:30). However, there were no significant changes in either actigraphy alpha or self‐reported sleep duration for the stable MCI pathway.

The dementia pathway showed a small‐to‐moderate increase in actigraphy alpha from ages 82 to 94 (−0.30 to −0.16), but no changes in actigraphy rest interval length. Notably, there were large within‐person increases in self‐reported duration of over an hour (06:22 to 07:31) on the dementia pathway; this level of change was significantly larger than those observed in either the normal or stable MCI path. Follow‐up exploratory analyses (Tables 4 and S5) showed that these gains became increasingly rapid as participants on the dementia pathway progressed from normal cognition (*d* [95% CI] = 0.146 [0.068, 0.223] from ages 82 to 86), to MCI (0.265 [0.075, 0.456] from ages 86.1 to 90), and finally to dementia (0.506 [0.185, 0.826] from 90.1 to 94).

#### 3.1.2 Regularity

All three pathways had decreasing regularity between ages 82 and 94, although in different indices and to varying degrees. The normal pathway exhibited a small increase in actigraphy midpoint variability (0.19−0.33), a small (non‐significant) decrease in IS (0.43−0.41) indicating less stability of RARs within each day, and a moderate increase in IV (1.19–1.38) indicating greater variability in RARs between days. The MCI pathway exhibited a small increase in actigraphy midpoint variability (0.17−0.40), a small (non‐significant) decrease in IS (0.46−0.43), and a large increase in IV (1.17–1.42). Finally, the dementia pathway had essentially no change in actigraphy midpoint variability, a small decrease in IS (0.45−0.40), and a large increase in IV (1.16–1.46).

Given the moderate‐to‐large effects for IV across pathways, we further investigated these changes along the key age segments. From ages 82 to 94, the stable MCI and dementia pathways had significantly larger increases in IV relative to the normal pathway. Exploratory follow‐up analyses (Tables 4 and S5) examining when these changes in IV occurred indicated that the three pathways had identical, small decreases in regularity (corresponding to increases in IV) during the initial period of normal cognition (*d* [95% CI] = 0.233 [0.204, 0.261] from ages 82 to 86). The normal pathway maintained this rate of change across follow‐up. IV increased at a faster rate after MCI diagnosis (*d* [95% CI] = 0.299 [0.232, 0.367] from ages 86.1 to 90) in the stable MCI pathway, and after both MCI (*d* [95% CI] = 0.278 [0.237, 0.319] from ages 86.1 to 90) and dementia diagnosis (*d* [95% CI] = 0.598 [0.359, 0.837] from ages 90.1 to 94) in the dementia pathway.

#### 3.1.3 Timing

All three pathways exhibited small‐to‐moderate shifts toward earlier objectively measured sleep timing between ages 82 and 94. The normal pathway exhibited moderate shifts toward earlier actigraphy midpoint (02:17−01:33) and small shifts toward earlier actigraphy acrophase (13:46–13:21). The stable MCI pathway exhibited small shifts toward both earlier actigraphy midpoint (02:14−01:37) and acrophase (13:52–13:18). The dementia pathway exhibited small advances in actigraphy midpoint (02:13−01:47) and moderate shifts toward earlier acrophase (13:54–13:13). Self‐reported sleep timing did not change meaningfully. Exploratory follow‐up analyses (Tables 4 and S5) suggested some evidence of greater advances during ages 90 to 94 in each cognitive pathway; however, the magnitude of these effects were considered very small or small, and inconsistencies were observed across self‐reported, actigraphy sleep, and actigraphy RAR measures.

### 3.2 Differences between cognitive pathways

Table 5 provides standardized between‐pathway differences and 95% CIs at age 82 (assuming normal cognition in all pathways) and at age 94 (assuming normal cognition for the normal pathway, MCI for the stable MCI pathway, and dementia for the dementia pathway). Below, we report the unstandardized results for each sleep/RAR outcome in its original scale, describe the magnitude of the effect size (very small, small, moderate, large), and summarize these findings by amount, regularity and duration.

**TABLE 5:** Standardized between‐pathway differences at benchmark ages 82 and 94.

	Age 82^a^			Age 94^b^		
	Normal vs. stable MCI	Normal vs. dementia	Stable MCI vs. dementia	Normal vs. stable MCI	Normal vs. dementia	Stable MCI vs. dementia
***Amount***						
SR duration	−0.030 (−0.227, −0.167)	**0.219 (0.044, 0.393)**	0.248 (0.019, 0.477)	−0.332 (−0.696, 0.033)	**−0.773 (–1.228, −0.318)**	−0.442 (−0.967, 0.084)
ACT rest interval length	0.063 (−0.022, 0.148)	**0.117 (0.044, 0.190)**	0.054 (−0.037, 0.145)	−0.146 (−0.696, 0.033)	−0.065 (−0.335, 0.204)	0.081(−0.235, 0.121)
Alpha	0.146 (−0.020, 0.312)	**0.179 (0.044, 0.315)**	0.034 (−0.143, 0.210)	0.235 (−0.112, 0.581)	−0.039 (−0.422, 0.345)	−0.273 (−0.748, 0.201)
***Regularity***						
Actigraphy midpoint SD	0.041 (−0.087, 0.170)	0.042 (−0.062, 0.146)	<0.001 (−0.138, 0.139)	−0.124 (−0.408, 0.160)	−0.191 (−0.113, 0.495)	0.315 (−0.057, 0.687)
Interdaily stability	**−0.235 (−0.408, −0.062)**	**−0.185 (−0.325, −0.045)**	0.050 (−0.134, 0.234)	−0.157 (−0.489, 0.175)	0.033 (−0.134, 0.234)	0.190 (−0.203, 0.582)
Intradaily variability	0.095 (−0.040, 0.230)	0.106 (−0.007, −0.220)	0.012 (−0.130, 0.153)	−0.172 (−0.444, 0.100)	**−0.304 (−0.585, −0.024)**	−0.132 (−0.496, 0.232)
***Timing***						
SR sleep midpoint	0.093 (−0.126, 0.312)	0.159 (−0.033, 0.352)	0.066 (−0.186, 0.319)	−0.038 (−0.377, 0.300)	0.233 (−0.160, 0.627)	0.272 (−0.184, 0.728)
ACT rest interval midpoint	0.042 (−0.118, 0.201)	0.050 (−0.079, 0.178)	0.008 (−0.164, 0.180)	−0.050 (−0.331, 0.230)	−0.175 (−0.479, 0.129)	−0.125 (−0.486, 0.236)
Acrophase	−0.080 (−0.269, 0.108)	−0.114 (−0.267, 0.040)	−0.033 (−0.232, 0.165)	0.039 (−0.297, 0.376)	0.109 (−0.253, 0.471)	0.070 (−0.376, 0.515)

*Notes*: Bold findings are significant after Benjamini–Hochberg multiple comparison adjustment within each feature type (amount, regularity, timing). Estimates are standardized such that values with magnitudes of |*d*| < 0.20 are considered very small, |*d*| ≥ 0.20−0.49 are considered small, |*d*| ≥ 0.50−0.79 are considered moderate, and |*d*| ≥ 0.80 are considered large.

Abbreviations: ACT, actigraphy; MCI, mild cognitive impairment; SR, self‐report.

^a^ Estimates are for “typical” participants at age 82 with average covariate levels. We assume no cognitive diagnoses in any of the three pathways.

^b^ Estimates are for “typical” participants at age 94 with average covariate levels. We assume no cognitive diagnosis on the normal pathway, MCI at age 86 on the stable MCI and dementia pathways, and dementia at age 90 on the dementia pathway.

#### 3.2.1 Amount

At age 82 (initial segment of normal cognition), the dementia pathway had significantly shorter self‐reported duration than the normal pathway (06:38 for normal, 06:22 for dementia) and the stable MCI pathway (06:40). These effect sizes were considered small, and the stable MCI–dementia comparison did not pass multiple comparison correction. The dementia pathway also had significantly shorter actigraphy rest interval length and alpha than the normal pathway at age 82; however, these effect sizes were all considered very small.

At age 94 (after cognitive diagnoses in the stable MCI and dementia pathways), the dementia pathway had significantly longer self‐reported sleep duration than participants on the normal pathway (07:31 for dementia, 06:33 for normal); this effect size was considered large. The dementia pathway also had longer self‐reported duration than the stable MCI pathway (06:58), with a moderate effect size; however, this finding was not statistically significant. There were no meaningful between‐pathway differences in actigraphy rest interval length or alpha at age 94.

#### 3.2.2 Regularity

At age 82, the normal pathway had lower IS than either the stable MCI pathway or the dementia pathway (0.43 for normal; 0.46 for stable MCI and 0.45 for dementia). However, the magnitudes of these IS differences were considered very small to small. No other regularity features differed between cognitive‐aging pathways at this age prior to diagnosis. After diagnosis at age 94, the dementia pathway had significantly greater IV than the normal pathway (1.46 for dementia; 1.38 for normal); the magnitude of this difference was small. Neither actigraphy midpoint SD nor IS differed significantly among groups at age 94.

#### 3.2.3 Timing

There were no significant between‐pathway differences in timing at age 82 or 94.

### 3.3 Sensitivity analyses

Findings from the four alternative sets of benchmark ages indicate that results are generally robust to this selection. Participants in the dementia pathway consistently had shorter self‐reported duration than participants in other pathways in the 3 to 4 years preceding an initial MCI diagnosis preceding dementia. Self‐reported duration increased with age on the dementia pathway, eventually overtaking the levels on the normal and stable MCI pathways after the onset of dementia. These trends were mirrored in actigraphy rest interval length, although the effect sizes were much smaller. Three of the four sets of benchmark ages showed small decreases in regularity (especially in dementia and MCI pathways). All four sets of benchmark ages showed a consistent trend toward earlier actigraphy midpoint and acrophase, regardless of the cognitive pathway. See Tables S6a–S9b in supporting information for detailed results.

## 4 DISCUSSION

This community‐based longitudinal study revealed self‐reported and actigraphy sleep and RAR features that differ within and across older adults who remain cognitively unimpaired, progress to stable MCI, or progress to dementia. During the initial period of normal cognition, those who ultimately received a dementia diagnosis reported shorter sleep than those with normal cognitive aging. Self‐reported sleep amount for participants on the dementia pathway increased at a faster rate relative to participants with normal cognitive aging, becoming roughly an hour longer than the other pathways after their dementia diagnosis. A similar pattern was observed for actigraphy rest interval length, although with much smaller effect sizes. These results suggest that a marked shift from short to long sleep duration among older adults may be a unique signature of cognitive decline and/or progressive neurodegeneration, rather than typical aging.

Although several studies have demonstrated that both short and long sleep are associated with greater risk for MCI and dementia,^51^, ^52^, ^53^ the precise explanation has been elusive.^54^, ^55^ Short sleep may directly increase risk for dementia by contributing to greater amyloid burden,^1^ exacerbating age‐related brain atrophy,^56^ or by increasing risk for other determinants of dementia (e.g., depression,^57^ cardiometabolic disease burden^58^). Alternatively, short sleep in the years prior to dementia may be a prodromal indicator rather than a risk factor.^59^ In contrast, longer sleep duration may reflect a compensatory response to existing neuropathology,^60^, ^61^, ^62^ including inflammation.^63^ Our findings extend this literature by showing temporal separation of associations between sleep amount and cognitive aging, with short sleep occurring prior to objective cognitive impairment and long sleep occurring after dementia diagnosis. This pattern was not observed among individuals who remain cognitively normal, consistent with prior cross‐sectional research.^64^ Overall, our findings may reflect the bidirectional nature of sleep–dementia associations over time, such that insufficient sleep may contribute to neuropathology associated with dementia, and progressive neuropathology may in turn interfere with sleep–wake processes.^5^, ^62^

We also observed changes in the regularity of sleep and RARs. During the initial period of normal cognition, all three pathways had similar initial levels and small increases in within‐day RAR fragmentation (i.e., IV). However, IV uniquely increased in the dementia pathway with MCI and dementia diagnoses. Similarly, the stability of between‐day RARs (i.e., IS) decreased in the dementia pathway, especially after dementia diagnosis, although this was not significant given the heterogeneity in the dementia pathway during this period. This heterogeneity may also explain the leveling effect of sleep midpoint variability in the dementia pathway after diagnosis, unlike in the other pathways. These observations are consistent with an earlier analysis conducted in MAP that found greater RAR fragmentation with age, accelerated rates of change in RARs with cognitive decline, and greater irregularity associated with incident dementia.^9^ In contrast, prospective analyses in a large cohort from the Netherlands did not reveal associations between 24 hour RARs measured at a single time point and subsequent risk of dementia.^10^ Our results add to the literature by suggesting that the *decreasing RAR regularity* may be a more useful index of cognitive decline and/or neurodegeneration than RAR levels alone.

The present study revealed a ubiquitous shift toward earlier actigraphy‐measured timing with age, regardless of the cognitive aging pathway, suggesting that age‐related changes in sleep homeostasis and circadian rhythms may explain these findings.^65^ However, we did not observe a similar trend for self‐reported midpoint. This discrepancy between measurement modalities may reflect heterogeneity in the accuracy of sleep–wake state perception or recollection when reported by individuals with varying degrees of cognitive impairment.^18^, ^66^ Indeed, a prior study conducted in cognitively healthy older adults found strong correlations between self‐reported and actigraphy‐derived measures of sleep amount but not for other sleep health indices.^16^ Nevertheless, our findings are in line with empirical research and clinical observations regarding sleep phase advances associated with both general aging and more prominently with dementia.^2^ Thus, changes in sleep timing alone may be less useful as a screening marker for dementia risk.

We observed some discrepancies in findings across measurement modalities (e.g., self‐reported/subjective vs. actigraphy‐derived/objective sleep and RARs), particularly related to sleep amount. While self‐reported total sleep time, actigraphy rest interval length, and actigraphy alpha all represent “sleep amount,” they inherently measure different features. Self‐reported total sleep time reflects perceived time spent asleep, while actigraphy rest interval length measures the entire time between sleep onset and offset, including periods of wakefulness. Objective–subjective discrepancies are also recognized across homologous measures, and may relate to insomnia, psychosocial factors, self‐rated health,^67^ sleep disorders,^68^ neuropathological burden,^69^ and genetic predispositions.^70^ While we adjusted for depressive symptoms and insomnia medication use, other factors not directly accounted for may partially explain the inconsistent results across measurement modalities. Nevertheless, such discrepancies suggest that subjective and objective sleep assessments may capture different mechanistic pathways in relation to cognitive health, highlighting the importance of comprehensive sleep health measurement.

While our objective was to characterize sleep health trajectories across cognitive aging pathways, clinical trials that manipulate the ART (amount, regularity, timing) of sleep health and evaluate cognitive and brain health outcomes are needed to determine whether sleep amount and regularity are modifiable causes of late‐life cognitive impairment and dementia. Prospective studies that assess sleep health earlier in the life course (e.g., in mid‐life) may be informative in determining causality or modifiability of associations between sleep health and cognitive decline or dementia. Better understanding of the timeline and causes of late‐life changes in sleep health and circadian rhythms can contribute to the development of interventions aimed at preventing MCI and dementia or delaying dementia progression. Even in the absence of clear evidence for sleep as a risk factor for dementia,^12^ better characterization of sleep health across normal and pathological cognitive aging can facilitate earlier detection and improve prognosis and treatment planning for older individuals with cognitive impairment.

### 4.1 Strengths, limitations, and future directions

This study has several notable strengths, including longitudinal measurement of sleep, RARs, and cognitive status. Our incorporation of both actigraphy‐derived and self‐reported sleep health allows for more nuanced interpretation, while also considering potential discrepancies among data types. The reliability of cognitive pathway assignment is strengthened by the multi‐step diagnostic process in Rush MAP,^31^ study criterion of two consecutive MCI diagnoses to establish age of MCI, and separation of individuals with stable MCI from those who progressed to dementia over time. We incorporated a comprehensive set of physical and mental health covariates as proxies for health comorbidities and an indicator of mortality during follow‐up to account for differential reasons for study dropout. Finally, we used a rigorous statistical approach allowing for flexible modeling of non‐linear sleep health trajectories and examined potential changes at meaningful intervals based on cognitive diagnosis.

Limitations of the present study must be addressed in future research. The Rush MAP study includes a geographically limited sample of participants who are primarily non‐Hispanic White, female, college educated, and consent to brain donation at study entry; thus, they may not be representative of the general population. The contrasts within and between the cognitive aging pathways were conducted using sample‐derived benchmark ages at MCI and dementia diagnosis. Although our findings were robust to the specific ages selected, replication in different samples (e.g., among septuagenarians, in other countries) will be important. External factors such as a participant's living environment (e.g., private home vs. assisted living facilities) not evaluated in the current study may also impact sleep and RARs beyond changes due to internal factors such as aging. Although our use of a stable MCI pathway facilitates a more homogenous dementia pathway, some people in this pathway may have been misclassified if they dropped out prior to dementia diagnosis. Moreover, the cognitive pathways defined in the present study assumed uni‐directional progressions, which may over‐simplify the complex nature of cognitive aging. Severe cognitive impairment may compromise the accuracy of self‐reported sleep data, and actigraphy‐based measures of sleep are approximations based on motor activity alone.^71^ Finally, our study focused on the sleep health domains that may be the most readily and directly modifiable through behavioral interventions. However, other aspects of sleep (e.g., sleep efficiency, sleep disorders, daytime napping) and RARs not measured in the study may also be informative and behaviorally modifiable.

### 4.2 Conclusions

This longitudinal study highlights differential changes in the amount and regularity of subjective and objective sleep between older adults who remain cognitively normal over time from those who go on to develop stable MCI or progress to dementia. These differences may help distinguish aspects of sleep health that are risk factors for future cognitive impairment or dementia from those that are consequences or prodromal indicators of dementia. This study also suggested that advances in sleep timing may be common across cognitive aging pathways, and thus less helpful for dementia screening. Additional experimental research is needed to understand the mechanisms underlying these findings, and to assess whether modifying sleep and RAR levels and trajectories can slow and/or delay pathological cognitive aging.

## CONFLICT OF INTEREST STATEMENT

Dr. Wallace reports grants from NIA RF1AG056331 and R01 AG083836, during the conduct of the study; personal fees from Health Rhythms, personal fees from Noctem Health, personal fees from Sleep Number Bed, outside the submitted work. Dr. Buysse has received grant/contract support from NIH, PCORI, AHRQ, VA, and Sleep Number for research relating to sleep, insomnia, sleep interventions, and questionnaire development. Dr. Buysse has served as a paid consultant to Sleep Number (not greater than $5000 per year). Consulting has focused on insomnia, measurement of sleep characteristics, and relationships between sleep and health outcomes. Dr. Buysse is an author of questionnaires including the Pittsburgh Sleep Quality Index, Pittsburgh Sleep Quality Index Addendum for PTSD (PSQI‐A), Brief Pittsburgh Sleep Quality Index (B‐PSQI), Daytime Insomnia Symptoms Scale, Pittsburgh Sleep Diary, Insomnia Symptom Questionnaire, and RU_SATED (copyrights held by University of Pittsburgh). These instruments have been licensed to commercial entities for fees by the University of Pittsburgh. Dr. Buysse receives a portion of the licensing fees, paid to him by the University of Pittsburgh. He is also co‐author of the Consensus Sleep Diary (copyright held by Ryerson University), which is licensed to commercial entities for a fee by Ryerson University. Dr. Buysse receives a portion of the licensing fees from the University of Pittsburgh through its agreement with Ryerson University. Dr. Zaheed is supported by the NHLBI (T32 HL082610; PI: Buysse). Dr. Leng is supported by NIA grants RF1AG056331 and R01AG083836. Author disclosures are available in the supporting information.

## CONSENT STATEMENT

The Memory and Aging Project (MAP) was approved by an institutional review board of Rush University Medical Center and all participants signed an informed consent, Anatomical Gift Act, and a repository consent to share data and biospecimens. All original data collection was performed in accordance with the ethical standards established in the 1964 Declaration of Helsinki and its later amendments. The grant facilitating secondary data analysis of MAP was declared exempt by the University of Pittsburgh Institutional Review Board (PRO17050218).

## Supporting information



Supporting Information

Supporting Information
