# Supplementary material for: Sleep trajectories across three cognitive‐aging pathways in community older adults
Source: Alzheimers Dement. 2025 May 2;21(5):e70159. doi: 10.1002/alz.70159 (PMC12046567; doi:10.1002/alz.70159)
Supplement: Supplementary file 2 — Supporting Information [file ALZ-21-e70159-s001.docx]

**Supplemental Materials**

Sleep trajectories across three cognitive-aging pathways in community older adults

Afsara B Zaheed*^1^, Amanda L Tapia*^1,2^, Nina Oryshkewych^1^, Bradley J Wheeler^3^, Meryl A Butters^1,4^, Daniel J Buysse^1,4^, Yue Leng^5^, Lisa L Barnes^6^, Andrew Lim^7^, Lan Yu^8^, Adriane M Soehner^1^, Meredith L Wallace^1,9^

*Joint first authors

^1^Department of Psychiatry, University of Pittsburgh, Pittsburgh PA, USA

^2^Department of Quantitative Health Sciences, Mayo Clinic, Rochester, MN, USA

^3^School of Computing and Information, University of Pittsburgh

^4^Clinical and Translational Science Institute, University of Pittsburgh

^5^Department of Psychiatry and Behavioral Sciences, University of California San Francisco, San Francisco, CA, USA

^6^Department of Neurological Sciences and Rush Alzheimer’s Disease Center, Rush University Medical Center, Chicago, IL, USA

^7^Department of Neurology, University of Toronto; Ontario, Canada

^8^Department of Medicine, University of Pittsburgh, Pittsburgh PA, USA

^9^Department of Statistics, University of Pittsburgh

Contents

[**Figure S1.** Flow chart depicting the derivation of the three final analytic samples. 3](#_Toc191745662)

[**Figure S2.** Illustration of cognitive aging pathways. 4](#_Toc191745663)

[**Figure S3.** Annotated figure for self-reported total sleep time 5](#_Toc191745664)

[**Table S1.** Demographic characteristics of the self-reported sleep, actigraphy sleep, and actigraphy rest-activity rhythm (RAR) samples. 6](#_Toc191745665)

[**Table S2**. Final cubic spline model parameters for sleep amount. 7](#_Toc191745666)

[**Table S3**. Final cubic spline model parameters for Regularity. 8](#_Toc191745667)

[**Table S4**. Final cubic spline model parameters for Timing. 9](#_Toc191745668)

[**Table S5.** Mean standardized changes (d) and 95% confidence intervals within each pathway, by age period. 10](#_Toc191745669)

[**Table S6a.** Sensitivity analysis 1: Estimated within-pathway changes and 95% Confidence Intervals in self-report sleep, RAR, and actigraphy sleep features. Estimates assume a starting age of 80 and end age of 92. 11](#_Toc191745670)

[**Table S6b.** Sensitivity analysis 1: Standardized between-pathway differences at benchmark ages 80 and 92. 11](#_Toc191745671)

[**Table S7a.** Sensitivity analysis 2: Estimated within-pathway changes and 95% Confidence Intervals (*d* [95% CI]) in self-report sleep, RAR, and actigraphy sleep features. Estimates assume a starting age of 84 and end age of 96. 12](#_Toc191745672)

[**Table S7b.** Sensitivity analysis 2: Standardized between-pathway differences at benchmark ages 84 and 96. 12](#_Toc191745673)

[**Table S8a.** Sensitivity analysis 3: Estimated within-pathway changes and 95% Confidence Intervals (*d* [95% CI]) in self-report sleep, RAR, and actigraphy sleep features. Estimates assume a starting age of 82 and end age of 91, with three-year intervals for each segment. 13](#_Toc191745674)

[**Table S8b.** Sensitivity analysis 3: Standardized between-pathway differences at benchmark ages 82 and 91. 13](#_Toc191745675)

[**Table S9a.** Sensitivity analysis 4: Estimated within-pathway changes and 95% Confidence Intervals (*d* [95% CI]) in self-report sleep, RAR, and actigraphy sleep features. Estimates assume a starting age of 80 and end age of 94. 14](#_Toc191745676)

[**Table S9b.** Sensitivity analysis 4: Standardized between-pathway differences at benchmark ages 80 and 94. 14](#_Toc191745677)

# **Figure S1.** Flow chart depicting the derivation of the three final analytic samples.

**
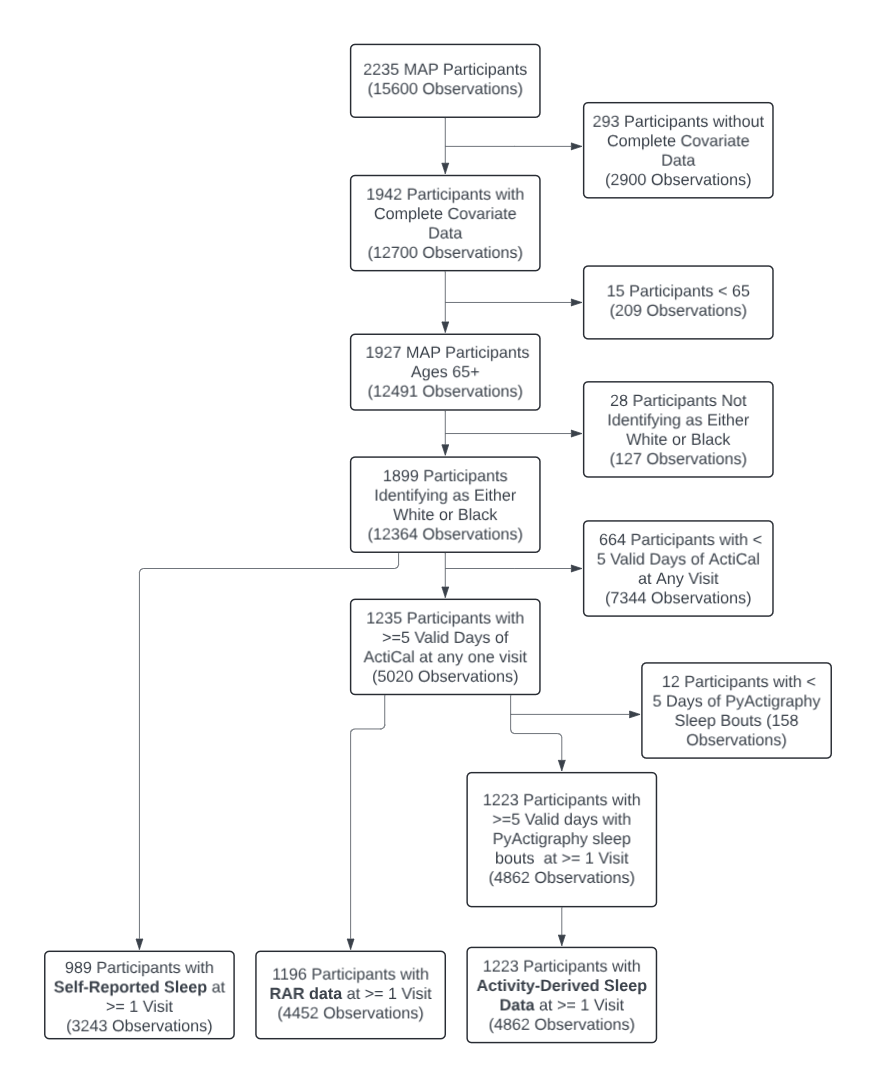
**

# **Figure S2.** Illustration of cognitive aging pathways.

**
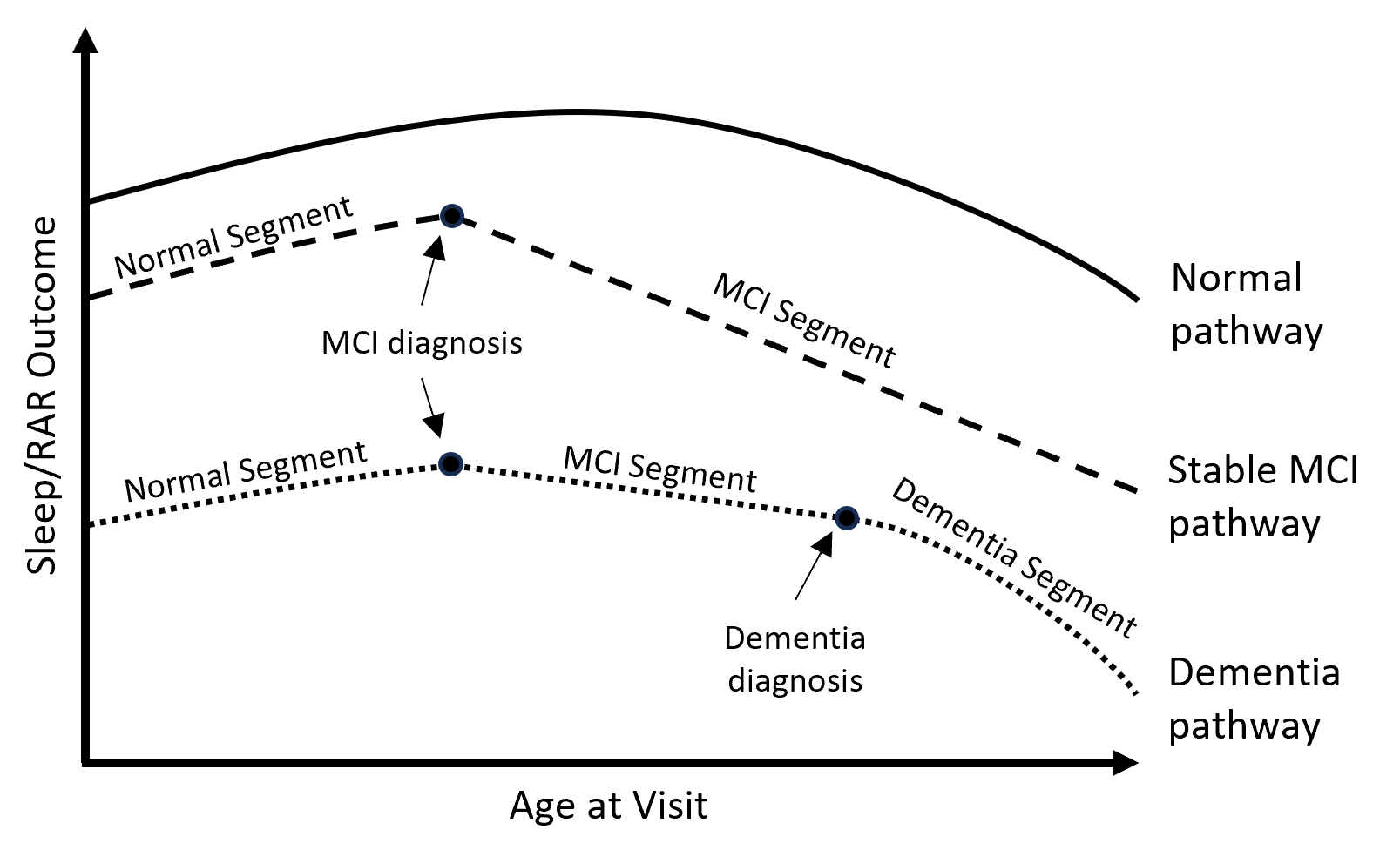
**

*Note*: Participants on the Normal pathway have only normal cognition spanning their entire follow-up. Participants on the Stable MCI pathway could contribute to a segment of normal cognition and/or a segment of MCI. For example, if a participant had a MCI diagnosis prior to their first sleep/RAR observation (and never dementia), they would contribute data only to the MCI segment of the Stable MCI pathway. Conversely, if a participant had normal cognition during their sleep/RAR observations, but a MCI diagnosis after the last sleep/RAR observation, they would contribute data only to the normal segment of the Stable MCI pathway. Similarly, participants on the Dementia pathway could contribute to a segment of normal cognition, MCI, and/or dementia. For example, if a participant had sleep/RAR data only during a period of normal cognition, then later went on to get MCI and dementia after their last sleep/RAR measurement, they would contribute data to the normal period of the Dementia pathway.

# **Figure S3.** Annotated figure for self-reported total sleep time


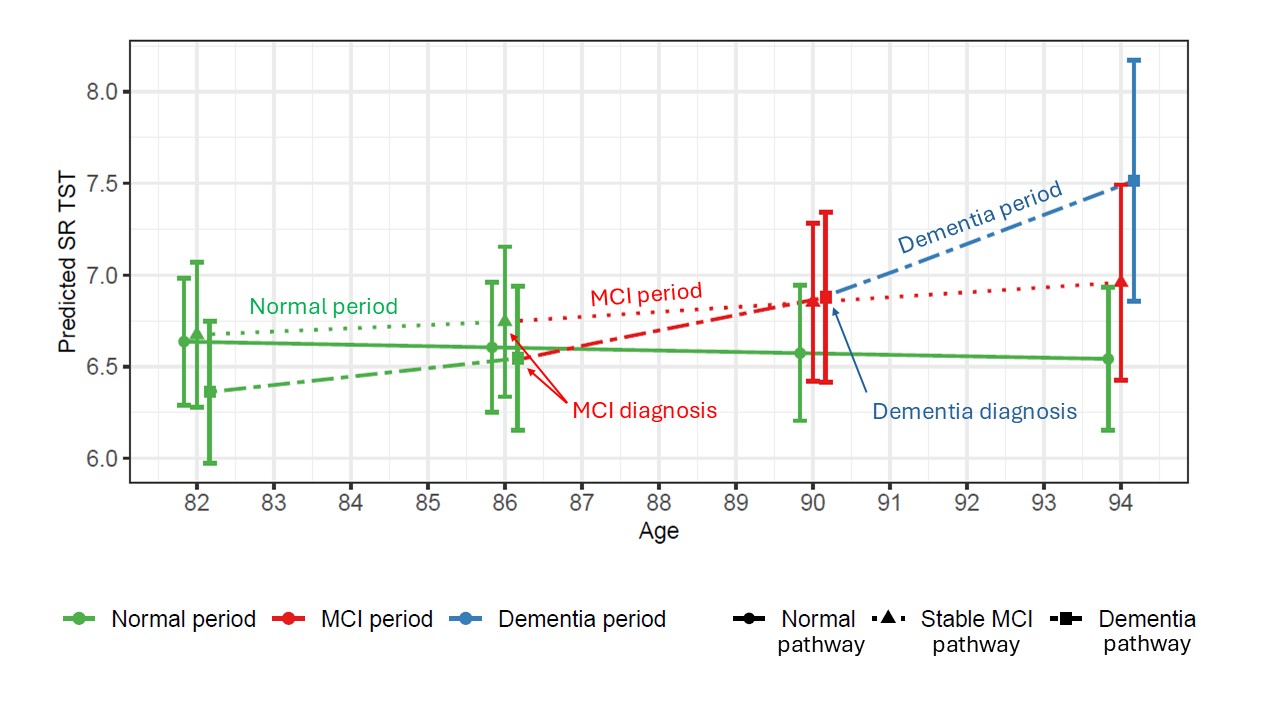


*Note:* Unstandardized self-reported (SR) total sleep time (TST; in hours) trajectories for Normal (circle markers), Stable Mild Cognitive Impairment (MCI; triangle markers), and Dementia (square markers) cognitive aging pathways. Green lines depict periods of normal cognition (i.e., no cognitive impairment) across all three pathways. Red lines depict periods of MCI in the Stable MCI and Dementia pathways. The Blue line depicts the dementia period in the Dementia pathway.

# **Table S1.** Demographic characteristics of the self-reported sleep, actigraphy sleep, and actigraphy rest-activity rhythm (RAR) samples.

|  | **Actigraphy RAR (N=1196)** | **Actigraphy Sleep**  **(N=1223)** | **Self-Reported Sleep (N=989)** |
| --- | --- | --- | --- |
| **Age at baseline**, Mean (SD) | 81.2 (7.09) | 81.2 (7.11) | 82.0 (7.37) |
| **Race**, N(%) |  |  |  |
| Black | 71 (5.9%) | 71 (5.8%) | 44 (4.4%) |
| White | 1125 (94.1%) | 1152 (94.2%) | 945 (95.6%) |
| **Sex**, N(%) |  |  |  |
| Female | 904 (75.6%) | 922 (75.4%) | 745 (75.3%) |
| Male | 292 (24.4%) | 301 (24.6%) | 244 (24.7%) |
| **Years of education,** Mean (SD) | 15.1 (3.01) | 15.1 (3.00) | 15.7 (3.03) |
| **Spanish/Hispanic/Latin Origin** |  |  |  |
| No | 1171 (97.9%) | 1197 (97.9%) | 970 (98.1%) |
| Yes | 25 (2.1%) | 26 (2.1%) | 19 (1.9%) |
| **Alcohol used per day (grams**), Mean (SD) | 5.53 (12.5) | 5.54 (12.5) | 6.13 (13.9) |
| **Early life socioeconomic status,** Mean (SD) | 0.0383 (0.720) | 0.0390 (0.717) | 0.134 (0.678) |
| **Died** |  |  |  |
| No | 537 (44.9%) | 543 (44.4%) | 639 (64.6%) |
| Yes | 659 (55.1%) | 680 (55.6%) | 350 (35.4%) |
| **Marital status** |  |  |  |
| Never married | 80 (6.7%) | 83 (6.8%) | 59 (6.0%) |
| Divorced/Separated | 138 (11.5%) | 135 (11.0%) | 128 (12.9%) |
| Married | 458 (38.3%) | 469 (38.3%) | 428 (43.3%) |
| Widowed | 520 (43.5%) | 536 (43.8%) | 374 (37.8%) |
| **Global cognitive function**, Mean(SD) | 0.0447 (0.654) | 0.0361 (0.667) | 0.153 (0.634) |
| **Medical conditions**, Mean(SD) | 1.53 (1.06) | 1.53 (1.07) | 1.60 (1.09) |
| **Instrumental activities of daily living**, Mean(SD) | 1.07 (1.65) | 1.08 (1.68) | 1.05 (1.64) |
| **Insomnia medications** |  |  |  |
| No | 1090 (91.1%) | 1115 (91.2%) | 910 (92.0%) |
| Yes | 106 (8.9%) | 108 (8.8%) | 79 (8.0%) |
| **CESD (sleep item removed)**, Mean(SD) | 0.908 (1.36) | 0.913 (1.37) | 0.815 (1.26) |
| **Age at 2 consecutive MCI dx (if applicable)**^a^ | 85.9 (6.02) | 85.8 (6.05) | 85.4 (6.29) |
| **Age at first dementia dx (if applicable)**^b^ | 88.4 (6.69) | 88.4 (6.70) | 88.5 (7.08) |
| **Years of follow-up after initial visit**, Mean(SD) | 3.59 (3.25) | 3.98 (2.75) | 3.28 (2.16) |
| **Cognitive pathway**, N(%) |  |  |  |
| Normal | 663 (55.4%) | 677 (55.4%) | 654 (66.1%) |
| Stable MCI | 166 (13.9%) | 169 (13.8%) | 125 (12.6%) |
| Dementia | 367 (30.7%) | 377 (30.8%) | 210 (21.2%) |

^a^ACT RAR: N=833 missing (69.6%); ACT Sleep: N=852 missing (69.7%); SR Sleep: N=768 missing (77.7%)

^b^ACT RAR: N=829 missing (69.3%); ACT Sleep: N=846 missing (69.2%); SR Sleep: N=779 missing (78.8%)

# **Table S2**. Final cubic spline model parameters for sleep amount.

| **Predictors** | **Self-Report TST** | **Actigraphy RIL** | **Alpha** |
| --- | --- | --- | --- |
| (Intercept) | 6.495 (6.010, 6.981) | 11.860 (11.588, 12.132) | -0.349 (-0.427, -0.271) |
| Age | -0.008 (-0.020, 0.004) | 0.001 (-0.004, 0.007) | 0.006 (0.004, 0.008) |
| MCI Path (vs. Normal) | -0.399 (-1.083, 0.285) | -0.074 (-0.175, 0.026) | -0.040 (-0.085, 0.006) |
| DEM Path (vs. Normal) | -1.187 (-1.723, -0.651) | -0.139 (-0.225, -0.052) | -0.049 (-0.086, -0.012) |
| Race (White vs. Black) | 0.949 (0.583, 1.314) | 0.063 (-0.066, 0.192) | -0.006 (-0.064, 0.052) |
| Sex (Male vs. Female) | -0.065 (-0.250, 0.120) | 0.076 (-0.001, 0.153) | 0.001 (-0.034, 0.036) |
| Education | 0.009 (-0.093, 0.112) | 0.063 (0.020, 0.107) | -0.008 (-0.027, 0.012) |
| Spanish Ethnicity (Yes vs. No) | -0.287 (-0.837, 0.262) | -0.164 (-0.379, 0.051) | 0.068 (-0.025, 0.162) |
| Alcohol Use | 0.047 (-0.015, 0.110) | -0.013 (-0.043, 0.017) | 0.003 (-0.009, 0.016) |
| Socioeconomic Status | 0.172 (0.053, 0.291) | -0.025 (-0.073, 0.022) | 0.004 (-0.017, 0.026) |
| Death | -0.021 (-0.191, 0.150) | -0.005 (-0.076, 0.065) | -0.031 (-0.062, -0.000) |
| Marital Status (Divorced/Separated vs. Never) | 0.081 (-0.273, 0.436) | 0.256 (0.112, 0.400) | 0.041 (-0.024, 0.105) |
| Marital Status (Married vs. Never) | 0.399 (0.079, 0.718) | 0.160 (0.034, 0.287) | -0.029 (-0.086, 0.028) |
| Marital Status (Widowed vs. Never) | 0.020 (-0.303, 0.343) | 0.127 (-0.003, 0.256) | -0.072 (-0.129, -0.015) |
| Global Cognition | -0.392 (-0.502, -0.282) | -0.119 (-0.178, -0.060) | -0.007 (-0.031, 0.016) |
| Medical Conditions | 0.012 (-0.046, 0.071) | 0.023 (-0.005, 0.052) | 0.016 (0.004, 0.028) |
| Instrumental Activities of Daily Living | 0.064 (0.022, 0.106) | 0.069 (0.042, 0.097) | 0.021 (0.011, 0.032) |
| Insomnia Diagnosis | -0.234 (-0.387, -0.081) | -0.111 (-0.207, -0.016) | -0.023 (-0.059, 0.014) |
| Depressive Symptoms (CESD) | -0.147 (-0.190, -0.104) | -0.007 (-0.035, 0.022) | 0.005 (-0.006, 0.016) |
| Dementia Spline (Dementia Path) | 0.076 (-0.022, 0.173) | -0.076 (-0.132, -0.019) | -0.011 (-0.030, 0.009) |
| MCI Spline (Dementia Path) | 0.038 (-0.024, 0.099) |  | 0.013 (0.001, 0.025) |
| MCI Spline (MCI Path) | 0.009 (-0.046, 0.064) |  | -0.003 (-0.015, 0.009) |
| Age x MCI Path | 0.026 (-0.009, 0.061) |  |  |
| Age x Dementia Path | 0.054 (0.028, 0.080) |  |  |
| MCI Spline (MCI Path)^3 |  | 0.000 (0.000, 0.001) |  |
| MCI Spline (Dementia Path)^3 |  | 0.001 (0.000, 0.002) |  |
| Percentage of Valid Sleep Days | NA | -3.946 (-4.171, -3.721) | NA |

*Note: TST = total sleep time and RIL = Rest Interval Length. Blank cells indicate that the feature was excluded as a result of the model-building procedure.*

# **Table S3**. Final cubic spline model parameters for Regularity.

| **Predictors** | **Actigraphy Midpoint SD** | **Interdaily Stability** | **Intradaily Variability** |
| --- | --- | --- | --- |
| (Intercept) | 2.174 (1.973, 2.375) | 0.446 (0.403, 0.489) | 0.890 (0.817, 0.962) |
| Age | -0.004 (-0.014, 0.007) | -0.002 (-0.006, 0.001) | 0.016 (0.014, 0.017) |
| Age^2 | 0.000 (0.000, 0.001) | 0.000 (-0.000, 0.000) |  |
| MCI Path (vs. Normal) | 0.087 (-0.125, 0.298) | 0.221 (0.120, 0.322) | -0.025 (-0.061, 0.011) |
| DEM Path (vs. Normal) | -0.024 (-0.178, 0.130) | -0.009 (-0.076, 0.058) | -0.028 (-0.059, 0.002) |
| Race (White vs. Black) | -0.149 (-0.252, -0.046) | 0.083 (0.056, 0.111) | 0.060 (0.006, 0.114) |
| Sex (Male vs. Female) | 0.029 (-0.026, 0.085) | -0.031 (-0.047, -0.016) | 0.067 (0.037, 0.097) |
| Education | -0.001 (-0.032, 0.031) | -0.016 (-0.025, -0.007) | 0.028 (0.012, 0.045) |
| Spanish Ethnicity (Yes vs. No) | 0.108 (-0.051, 0.267) | -0.002 (-0.045, 0.041) | -0.006 (-0.091, 0.079) |
| Alcohol Use | -0.026 (-0.046, -0.006) | 0.002 (-0.003, 0.008) | -0.010 (-0.021, 0.000) |
| Socioeconomic Status | 0.027 (-0.007, 0.062) | -0.002 (-0.011, 0.007) | 0.034 (0.016, 0.052) |
| Death | -0.055 (-0.106, -0.005) | 0.031 (0.017, 0.044) | -0.023 (-0.049, 0.004) |
| Marital Status (Divorced/Separated vs. Never) | 0.103 (-0.005, 0.210) | -0.047 (-0.077, -0.018) | 0.026 (-0.032, 0.084) |
| Marital Status (Married vs. Never) | 0.092 (-0.000, 0.185) | -0.024 (-0.049, 0.002) | 0.015 (-0.035, 0.065) |
| Marital Status (Widowed vs. Never) | 0.081 (-0.010, 0.172) | -0.028 (-0.053, -0.003) | -0.009 (-0.058, 0.040) |
| Global Cognition | -0.014 (-0.054, 0.025) | 0.012 (0.003, 0.021) | -0.027 (-0.045, -0.009) |
| Medical Conditions | 0.039 (0.020, 0.058) | -0.010 (-0.015, -0.006) | 0.014 (0.004, 0.024) |
| Instrumental Activities of Daily Living | 0.081 (0.063, 0.099) | -0.009 (-0.013, -0.006) | 0.052 (0.044, 0.059) |
| Insomnia Diagnosis | -0.038 (-0.102, 0.026) | 0.009 (-0.004, 0.022) | -0.044 (-0.071, -0.017) |
| Depressive Symptoms (CESD) | 0.015 (-0.004, 0.034) | -0.005 (-0.009, -0.002) | 0.006 (-0.002, 0.014) |
| Percentage of Valid Sleep Days | -2.236 (-2.381, -2.091) |  |  |
| MCI Spline (MCI Path) | 0.022 (0.001, 0.043) | -0.004 (-0.009, 0.000) |  |
| MCI Spline (Dementia Path) | -0.005 (-0.026, 0.015) | 0.002 (-0.002, 0.007) |  |
| Dementia Spline (Dementia Path) | -0.011 (-0.044, 0.023) | -0.003 (-0.010, 0.004) | 0.003 (-0.011, 0.018) |
| Age x MCI Path | -0.007 (-0.017, 0.004) | -0.019 (-0.029, -0.009) |  |
| Age x Dementia Path | -0.000 (-0.007, 0.007) | 0.005 (-0.002, 0.012) |  |
| Age^2 x MCI Path |  | 0.000 (0.000, 0.001) |  |
| Age^2 x Dementia Path |  | -0.000 (-0.000, -0.000) |  |
| MCI Spline (Dementia Path)^3 |  |  | 0.000 (0.000, 0.000) |
| MCI Spline (MCI Path)^2 |  |  | 0.001 (0.000, 0.002) |

*Note: Blank cells indicate that the feature was excluded as a result of the model-building procedure.*

# **Table S4**. Final cubic spline model parameters for Timing.

| **Predictors** | **Self-Report Midpoint** | **Actigraphy Midpoint** | **Acrophase** |
| --- | --- | --- | --- |
| (Intercept) | 26.635 (26.162, 27.107) | 22.947 (22.433, 23.461) | 14.169 (13.727, 14.611) |
| Age | -0.046 (-0.091, -0.001) | -0.006 (-0.032, 0.020) | -0.034 (-0.044, -0.023) |
| Age^2 | 0.004 (0.001, 0.006) | -0.001 (-0.002, -0.000) |  |
| Age^3 | -0.000 (-0.000, -0.000) |  |  |
| MCI Path (vs. Normal) | 0.564 (-0.653, 1.782) | -0.039 (-0.616, 0.539) | 0.101 (-0.137, 0.339) |
| DEM Path (vs. Normal) | -0.361 (-1.151, 0.429) | -0.349 (-0.760, 0.062) | 0.143 (-0.050, 0.337) |
| Race (White vs. Black) | 0.017 (-0.295, 0.330) | -0.168 (-0.474, 0.139) | 0.120 (-0.213, 0.454) |
| Sex (Male vs. Female) | 0.091 (-0.061, 0.242) | -0.038 (-0.210, 0.134) | -0.275 (-0.467, -0.084) |
| Education | 0.029 (-0.054, 0.113) | 0.096 (0.000, 0.192) | 0.082 (-0.024, 0.188) |
| Spanish Ethnicity (Yes vs. No) | -0.650 (-1.106, -0.195) | -0.158 (-0.629, 0.314) | -0.472 (-0.997, 0.054) |
| Alcohol Use | -0.015 (-0.067, 0.038) | -0.017 (-0.079, 0.046) | 0.010 (-0.058, 0.079) |
| Socioeconomic Status | 0.048 (-0.049, 0.144) | -0.110 (-0.215, -0.005) | -0.025 (-0.140, 0.091) |
| Death | 0.068 (-0.066, 0.201) | 0.278 (0.126, 0.430) | 0.225 (0.057, 0.394) |
| Marital Status (Divorced/Separated vs. Never) | 0.327 (0.026, 0.628) | 0.208 (-0.122, 0.539) | 0.310 (-0.054, 0.675) |
| Marital Status (Married vs. Never) | 0.184 (-0.085, 0.453) | 0.250 (-0.035, 0.535) | 0.469 (0.151, 0.786) |
| Marital Status (Widowed vs. Never) | 0.177 (-0.090, 0.445) | 0.374 (0.094, 0.653) | 0.484 (0.172, 0.795) |
| Global Cognition | 0.054 (-0.017, 0.126) | -0.089 (-0.183, 0.005) | -0.087 (-0.197, 0.024) |
| Medical Conditions | 0.060 (0.018, 0.101) | 0.057 (0.003, 0.110) | 0.049 (-0.011, 0.109) |
| Instrumental Activities of Daily Living | -0.053 (-0.079, -0.027) | 0.098 (0.059, 0.138) | -0.031 (-0.076, 0.015) |
| Insomnia Diagnosis | 0.014 (-0.081, 0.108) | 0.146 (0.006, 0.285) | 0.124 (-0.040, 0.287) |
| Depressive Symptoms (CESD) | 0.016 (-0.010, 0.043) | 0.033 (-0.008, 0.074) | 0.017 (-0.031, 0.065) |
| MCI Spline (Dementia Path) | 0.018 (-0.023, 0.058) | -0.022 (-0.068, 0.025) | -0.015 (-0.067, 0.036) |
| Dementia Spline (Dementia Path)^2 | -0.012 (-0.024, -0.000) |  |  |
| MCI Spline (MCI Path) | 0.011 (-0.027, 0.049) | 0.018 (-0.032, 0.067) | -0.019 (-0.070, 0.033) |
| Age x MCI Path | -0.063 (-0.179, 0.053) | -0.001 (-0.029, 0.027) |  |
| Age x Dementia Path | 0.020 (-0.051, 0.092) | 0.016 (-0.003, 0.036) |  |
| Age^2 x MCI Path | 0.001 (-0.001, 0.004) |  |  |
| Age^2 x Dementia Path | -0.000 (-0.002, 0.001) |  |  |
| Percentage of Valid Sleep Days |  | 3.869 (3.569, 4.169) |  |
| Dementia Spline (Dementia Path) |  | 0.072 (-0.003, 0.147) | -0.040 (-0.123, 0.043) |

*Note: Blank cells indicate that the feature was excluded as a result of the model-building procedure.*

# **Table S5.** Mean standardized changes (d) and 95% confidence intervals within each pathway, by age period.

| **Feature** | **Normal Path**^a^ | **Stable MCI Path**^b^ | **Dementia Path**^c^ |
| --- | --- | --- | --- |
| *Initial Period of Normal Cognition (Ages 82-86)^e^* | | | |
| Self-reported Total Sleep Time | -0.025 (-0.064, 0.014) | 0.057 (-0.051, 0.164) | **0.146 (0.068, 0.223)** |
| Actigraphy Rest Interval Length | 0.004 (-0.014, 0.023) | 0.004 (-0.014, 0.023) | 0.004 (-0.014, 0.023) |
| Alpha | **0.089 (0.057, 0.120)** | **0.089 (0.057, 0.120)** | **0.089 (0.057, 0.120)** |
| Actigraphy Midpoint Variability | **0.059 (0.026, 0.091)** | 0.014 (-0.051, 0.080) | **0.058 (0.017, 0.100)** |
| Interdaily Stability | **-0.07 (-0.109, -0.031)** | **-0.127 (-0.206, -0.047)** | **-0.131 (-0.183, -0.080)** |
| Intradaily Variability | **0.233 (0.204, 0.261)** | **0.233 (0.204, 0.261)** | **0.233 (0.204, 0.261)** |
| Self-reported Midpoint | 0.042 (-0.004, 0.088) | 0.006 (-0.105, 0.117) | 0.052 (-0.033, 0.138) |
| Actigraphy Rest Interval Midpoint | **-0.150 (-0.186, -0.115)** | **-0.153 (-0.230, -0.076)** | **-0.103 (-0.151, -.054)** |
| Acrophase | **-0.107 (-0.141, -0.073)** | **-0.107 (-0.141, -0.073)** | **-0.107 (-0.141, -0.073)** |
| *Period of MCI (Ages 86 – 90) ^e^* | | | |
| Self-reported Total Sleep Time | -0.025 (-0.064, 0.014) | 0.085 (-0.075, 0.245) | **0.265 (0.075, 0.456)** |
| Actigraphy Rest Interval Length | 0.004 (-0.014, 0.023) | **0.031 (0.002, 0.059)** | **0.059 (0.022, 0.097)** |
| Alpha | **0.089 (0.057, 0.120)** | 0.044 (-0.132, 0.220) | **0.276 (0.101, 0.450)** |
| Actigraphy Midpoint Variability | **0.076 (0.033, 0.119)** | **0.181 (0.040, 0.322)** | 0.039 (-0.096, 0.173) |
| Interdaily Stability | **-0.068 (-0.124, -0.012)** | -0.138 (-0.287, 0.011) | -0.094 (-0.230, 0.043) |
| Intradaily Variability | **0.233 (0.204, 0.261)** | **0.299 (0.232, 0.367)** | **0.278 (0.237, 0.319)** |
| Self-reported Midpoint | 0.004 (-0.049, 0.057) | 0.063 (-0.078, 0.204) | 0.073 (-0.089, 0.234) |
| Actigraphy Rest Interval Midpoint | **-0.178 (-0.224, -0.132)** | **-0.131 (-0.258, -0.004)** | **-0.193 (-0.324, -0.063)** |
| Acrophase | **-0.107 (-0.141, -0.073)** | **-0.166 (-0.328, -0.004)** | -0.155 (-0.317, 0.007) |
| *Period of Dementia (Ages 90 – 94)* | | | |
| Self-reported Total Sleep Time | -0.025 (-0.064, 0.014) | 0.085 (-0.075, 0.245) | **0.506 (0.185, 0.826)** |
| Actigraphy Rest Interval Length | 0.004 (-0.014, 0.023) | **0.187 (0.027, 0.347)** | 0.132 (-0.107, 0.371) |
| Alpha | **0.089 (0.057, 0.120)** | 0.044 (-0.132, 0.220) | 0.120 (-0.158, 0.398) |
| Actigraphy Midpoint Variability | **0.094 (0.037, 0.150)** | **0.199 (0.055, 0.342)** | -0.018 (-0.243, 0.207) |
| Interdaily Stability | -0.067 (-0.143, 0.010) | -0.018 (-0.196, 0.160) | -0.197 (-0.418, 0.024) |
| Intradaily Variability | **0.233 (0.204, 0.261)** | **0.433 (0.240, 0.626)** | **0.598 (0.359, 0.837)** |
| Self-reported Midpoint | -0.065 (-0.152, 0.021) | 0.043 (-0.142, 0.227) | -0.219 (-0.465, 0.028) |
| Actigraphy Rest Interval Midpoint | **-0.206 (-0.265, -0.147)** | **-0.159 (-0.290, -0.028)** | -0.013 (-0.226, 0.199) |
| Acrophase | **-0.107 (-0.141, -0.073)** | **-0.166 (-0.328, -0.004)** | **-0.281 (-0.536, -0.026)** |

**^a^**Estimates are for a ‘typical’ participant on the Normal pathway, with no cognitive diagnosis across follow-up and average levels of covariates.

**^b^**Estimates are for a ‘typical’ participant on the Stable MCI path, with MCI at age 86 and no dementia diagnosis.

**^c^**Estimates are for a ‘typical’ participant on the Dementia path, with MCI at age 86 and a dementia diagnosis at age 90.

*Note: Bold figures are statistically significant.*

# **Table S6a.** Sensitivity analysis 1: Estimated within-pathway changes and 95% Confidence Intervals in self-report sleep, RAR, and actigraphy sleep features. Estimates assume a starting age of 80 and end age of 92.

| **Feature** | **Normal Path**^a^ | **Stable MCI Path**^b^ | **Dementia Path**^c^ |
| --- | --- | --- | --- |
| *Amount* | | | |
| Self-reported Total Sleep Time | -0.094 (-0.242, 0.053) | 0.285 (-0.164, 0.734) | **1.152 (0.595, 1.709)** |
| Actigraphy Rest Interval Length | 0.016 (-0.049, 0.081) | **0.263 (0.041, 0.485)** | 0.232 (-0.084, 0.547) |
| Alpha | **0.073 (0.047, 0.098)** | 0.048 (-0.049, 0.145) | 0.132 (0.03, 0.234) |
| *Regularity* | | | |
| Actigraphy Midpoint St.Dev. | **0.119 (0.054, 0.184)** | **0.217 (0.04, 0.393)** | 0.031 (-0.143, 0.205) |
| Interdaily Stability | **-0.023 (-0.042, -0.005)** | **-0.053 (-0.093, -0.013)** | **-0.046 (-0.083, -0.008)** |
| Intradaily Variability | **0.186 (0.164, 0.209)** | **0.258 (0.187, 0.328)** | **0.296 (0.225, 0.368)** |
| *Timing* | | | |
| Self-reported Midpoint | 0.046 (-0.084, 0.176) | 0.102 (-0.19, 0.395) | -0.001 (-0.367, 0.365) |
| Actigraphy Rest Interval Midpoint | **-0.682 (-0.848, -0.516)** | **-0.556 (-0.93, -0.181)** | -0.371 (-0.761, 0.019) |
| Acrophase | **-0.404 (-0.533, -0.275)** | **-0.554 (-0.969, -0.14)** | **-0.685 (-1.118, -0.252)** |

**^a^**Estimates are for a ‘typical’ participant on the Normal pathway, with no cognitive diagnosis across follow-up and average levels of covariates.

**^b^**Estimates are for a ‘typical’ participant on the Stable MCI path, with MCI at age 84 and no dementia diagnosis.

**^c^**Estimates are for a ‘typical’ participant on the Dementia path, with MCI at age 84 and a dementia diagnosis at age 88.

*Note:* *Bold figures are statistically significant.*

# **Table S6b.** Sensitivity analysis 1: Standardized between-pathway differences at benchmark ages 80 and 92.

| **Age 80**^a^ | | | |
| --- | --- | --- | --- |
| **Feature** | **MCI vs. Normal** | **Dementia vs. Normal** | **Dementia vs. MCI** |
| Self-Report Total Sleep Time | -0.014 (-0.284, 0.255) | **-0.382 (-0.618, -0.146)** | **-0.368 (-0.687, -0.049)** |
| Actigraphy Rest Interval Length | -0.074 (-0.175, 0.026) | -0.139 (-0.225, -0.052) | -0.064 (-0.171, 0.043) |
| Alpha | -0.04 (-0.085, 0.006) | -0.049 (-0.086, -0.012) | -0.009 (-0.057, 0.039) |
| Actigraphy Midpoint SD | -0.011 (-0.096, 0.073) | -0.024 (-0.091, 0.042) | -0.013 (-0.105, 0.079) |
| Interdaily Stability | **0.036 (0.014, 0.058)** | **0.024 (0.007, 0.042)** | -0.012 (-0.035, 0.012) |
| Intradaily Variability | -0.025 (-0.061, 0.011) | -0.028 (-0.059, 0.002) | -0.003 (-0.041, 0.035) |
| Self-Report Sleep Midpoint | -0.054 (-0.282, 0.174) | -0.161 (-0.36, 0.038) | -0.107 (-0.376, 0.162) |
| Actigraphy Midpoint | -0.055 (-0.297, 0.187) | -0.102 (-0.292, 0.088) | -0.046 (-0.310, 0.217) |
| Acrophase | 0.101 (-0.137, 0.339) | 0.143 (-0.05, 0.337) | 0.042 (-0.209, 0.293) |
| **Age 92**^b^ | | | |
| **Feature** | **MCI vs. Normal** | **Dementia vs. Normal** | **Dementia vs. MCI** |
| Self-Report Total Sleep Time | 0.365 (-0.068, 0.798) | **0.864 (0.301, 1.427)** | 0.499 (-0.146, 1.143) |
| Actigraphy Rest Interval Length | 0.173 (-0.053, 0.398) | 0.077 (-0.242, 0.396) | -0.095 (-0.469, 0.278) |
| Alpha | -0.064 (-0.159, 0.03) | 0.011 (-0.094, 0.115) | 0.075 (-0.055, 0.204) |
| Actigraphy Midpoint SD | 0.086 (-0.075, 0.247) | -0.112 (-0.289, 0.064) | -0.198 (-0.415, 0.018) |
| Interdaily Stability | 0.006 (-0.033, 0.045) | 0.002 (-0.038, 0.041) | -0.004 (-0.053, 0.044) |
| Intradaily Variability | 0.046 (-0.027, 0.119) | 0.081 (0.006, 0.156) | 0.035 (-0.062, 0.133) |
| Self-Report Sleep Midpoint | 0.002 (-0.287, 0.29) | -0.208 (-0.571, 0.154) | -0.21 (-0.627, 0.207) |
| Actigraphy Midpoint | 0.072 (-0.305, 0.449) | 0.21 (-0.204, 0.624) | 0.139 (-0.359, 0.636) |
| Acrophase | -0.049 (-0.474, 0.376) | -0.138 (-0.595, 0.319) | -0.088 (-0.651, 0.474) |

^a^Assumes that all pathways have normal cognition. Estimates are adjusted for average levels of all covariates.

^b^Assumes that participants on the Dementia pathway have dementia, participants on the Stable MCI pathway have MCI, and participants on the Normal pathway have no cognitive diagnosis. Estimates are adjusted for average levels of all covariates.

*Note: Bold figures are statistically significant.*

# **Table S7a.** Sensitivity analysis 2: Estimated within-pathway changes and 95% Confidence Intervals (*d* [95% CI]) in self-report sleep, RAR, and actigraphy sleep features. Estimates assume a starting age of 84 and end age of 96.

| **Feature** | **Normal Path**^a^ | **Stable MCI Path**^b^ | **Dementia Path**^c^ |
| --- | --- | --- | --- |
| *Amount* | | | |
| Self-reported Total Sleep Time | -0.094 (-0.242, 0.053) | 0.285 (-0.164, 0.734) | **1.152 (0.595, 1.709)** |
| Actigraphy Rest Interval Length | 0.016 (-0.049, 0.081) | 0.263 (0.041, 0.485) | 0.232 (-0.084, 0.547) |
| Alpha | 0.073 (0.047, 0.098) | 0.048 (-0.049, 0.145) | 0.132 (0.03, 0.234) |
| *Regularity* | | | |
| Actigraphy Midpoint St.Dev. | **0.150 (0.063, 0.237)** | **0.247 (0.066, 0.428)** | 0.062 (-0.115, 0.239) |
| Interdaily Stability | -0.022 (-0.048, 0.004) | -0.007 (-0.057, 0.044) | -0.062 (-0.102, -0.022) |
| Intradaily Variability | **0.186 (0.164, 0.209)** | **0.258 (0.187, 0.328)** | **0.296 (0.225, 0.368)** |
| *Timing* | | | |
| Self-reported Midpoint | -0.105 (-0.299, 0.089) | 0.087 (-0.293, 0.468) | -0.197 (-0.562, 0.167) |
| Actigraphy Rest Interval Midpoint | **-0.799 (-1.016, -0.582)** | **-0.672 (-1.061, -0.282)** | **-0.487 (-0.886, -0.088)** |
| Acrophase | **-0.404 (-0.533, -0.275)** | **-0.554 (-0.969, -0.14)** | **-0.685 (-1.118, -0.252)** |

**^a^**Estimates are for a ‘typical’ participant on the Normal pathway, with no cognitive diagnosis across follow-up and average levels of covariates.

**^b^**Estimates are for a ‘typical’ participant on the Stable MCI path, with MCI at age 88 and no dementia diagnosis.

**^c^**Estimates are for a ‘typical’ participant on the Dementia path, with MCI at age 88 and a dementia diagnosis at age 92.

*Note: Bold figures are statistically significant.*

# **Table S7b.** Sensitivity analysis 2: Standardized between-pathway differences at benchmark ages 84 and 96.

| **Age 84**^a^ | | | |
| --- | --- | --- | --- |
| **Feature** | **MCI vs. Normal** | **Dementia vs. Normal** | **Dementia vs. MCI** |
| Self-Report Total Sleep Time | 0.088 (-0.156, 0.333) | -0.168 (-0.382, 0.047) | -0.256 (-0.533, 0.021) |
| Actigraphy Rest Interval Length | -0.074 (-0.175, 0.026) | **-0.139 (-0.225, -0.052)** | -0.064 (-0.171, 0.043) |
| Alpha | -0.04 (-0.085, 0.006) | -0.049 (-0.086, -0.012) | -0.009 (-0.057, 0.039) |
| Actigraphy Midpoint SD | -0.037 (-0.109, 0.034) | -0.025 (-0.084, 0.034) | 0.013 (-0.063, 0.089) |
| Interdaily Stability | **0.022 (0.002, 0.043)** | **0.02 (0.003, 0.036)** | -0.003 (-0.024, 0.019) |
| Intradaily Variability | -0.025 (-0.061, 0.011) | -0.028 (-0.059, 0.002) | -0.003 (-0.041, 0.035) |
| Self-Report Sleep Midpoint | -0.111 (-0.312, 0.091) | -0.144 (-0.317, 0.03) | -0.033 (-0.26, 0.194) |
| Actigraphy Midpoint | -0.06 (-0.273, 0.154) | -0.036 (-0.21, 0.139) | 0.024 (-0.202, 0.25) |
| Acrophase | 0.101 (-0.137, 0.339) | 0.143 (-0.05, 0.337) | 0.042 (-0.209, 0.293) |
| **Age 96**^b^ | | | |
| **Feature** | **MCI vs. Normal** | **Dementia vs. Normal** | **Dementia vs. MCI** |
| Self-Report Total Sleep Time | 0.468 (-0.023, 0.959) | **1.079 (0.493, 1.665)** | 0.611 (-0.073, 1.295) |
| Actigraphy Rest Interval Length | 0.173 (-0.053, 0.398) | 0.077 (-0.242, 0.396) | -0.095 (-0.469, 0.278) |
| Alpha | -0.064 (-0.159, 0.03) | 0.011 (-0.094, 0.115) | 0.075 (-0.055, 0.204) |
| Actigraphy Midpoint SD | 0.06 (-0.116, 0.235) | -0.113 (-0.295, 0.07) | -0.172 (-0.396, 0.051) |
| Interdaily Stability | 0.038 (-0.014, 0.09) | -0.02 (-0.065, 0.026) | -0.058 (-0.115, 0.000) |
| Intradaily Variability | 0.046 (-0.027, 0.119) | **0.081 (0.006, 0.156)** | 0.035 (-0.062, 0.133) |
| Self-Report Sleep Midpoint | 0.082 (-0.305, 0.469) | -0.236 (-0.631, 0.159) | -0.318 (-0.794, 0.159) |
| Actigraphy Midpoint | 0.067 (-0.341, 0.475) | 0.276 (-0.156, 0.708) | 0.209 (-0.301, 0.719) |
| Acrophase | -0.049 (-0.474, 0.376) | -0.138 (-0.595, 0.319) | -0.088 (-0.651, 0.474) |

^a^Assumes that all pathways have normal cognition. Estimates are adjusted for average levels of all covariates.

^b^Assumes that participants on the Dementia pathway have dementia, participants on the Stable MCI pathway have MCI, and participants on the Normal pathway have no cognitive diagnosis. Estimates are adjusted for average levels of all covariates.

*Note: Bold figures are statistically significant.*

# **Table S8a.** Sensitivity analysis 3: Estimated within-pathway changes and 95% Confidence Intervals (*d* [95% CI]) in self-report sleep, RAR, and actigraphy sleep features. Estimates assume a starting age of 82 and end age of 91, with three-year intervals for each segment.

| **Feature** | **Normal Path**^a^ | **Stable MCI Path**^b^ | **Dementia Path**^c^ |
| --- | --- | --- | --- |
| *Amount* | | | |
| Self-reported Total Sleep Time | -0.071 (-0.181, 0.04) | 0.214 (-0.123, 0.551) | **0.864 (0.446, 1.282)** |
| Actigraphy Rest Interval Length | 0.012 (-0.037, 0.06) | **0.116 (0.015, 0.217)** | 0.004 (-0.174, 0.181) |
| Alpha | **0.054 (0.035, 0.074)** | 0.036 (-0.037, 0.109) | **0.099 (0.023, 0.175)** |
| *Regularity* | | | |
| Actigraphy Midpoint St.Dev. | 0.092 (0.042, 0.143) | **0.165 (0.033, 0.298)** | 0.026 (-0.104, 0.157) |
| Interdaily Stability | -0.017 (-0.032, -0.003) | -0.036 (-0.066, -0.005) | **-0.036 (-0.064, -0.008)** |
| Intradaily Variability | 0.140 (0.123, 0.157) | **0.180 (0.139, 0.221)** | 0.190 (0.146, 0.234) |
| *Timing* | | | |
| Self-reported Midpoint | 0.035 (-0.066, 0.136) | 0.090 (-0.131, 0.31) | 0.032 (-0.228, 0.292) |
| Actigraphy Rest Interval Midpoint | **-0.523 (-0.651, -0.394)** | **-0.428 (-0.709, -0.146)** | -0.289 (-0.582, 0.004) |
| Acrophase | **-0.303 (-0.399, -0.206)** | **-0.416 (-0.727, -0.105)** | -0.514 (-0.838, -0.189) |

**^a^**Estimates are for a ‘typical’ participant on the Normal pathway, with no cognitive diagnosis across follow-up and average levels of covariates.

**^b^**Estimates are for a ‘typical’ participant on the Stable MCI path, with MCI at age 85 and no dementia diagnosis.

**^c^**Estimates are for a ‘typical’ participant on the Dementia path, with MCI at age 85 and a dementia diagnosis at age 88.

*Note: Bold figures are statistically significant.*

# **Table S8b.** Sensitivity analysis 3: Standardized between-pathway differences at benchmark ages 82 and 91.

| **Age 82**^a^ | | | |
| --- | --- | --- | --- |
| **Feature** | **MCI vs. Normal** | **Dementia vs. Normal** | **Dementia vs. MCI** |
| Self-Report Total Sleep Time | 0.037 (-0.21, 0.285) | **-0.275 (-0.494, -0.056)** | **-0.312 (-0.6, -0.024)** |
| Actigraphy Rest Interval Length | -0.074 (-0.175, 0.026) | -0.139 (-0.225, -0.052) | -0.064 (-0.171, 0.043) |
| Alpha | -0.04 (-0.085, 0.006) | -0.049 (-0.086, -0.012) | -0.009 (-0.057, 0.039) |
| Actigraphy Midpoint SD | -0.024 (-0.1, 0.051) | -0.025 (-0.086, 0.037) | 0 (-0.082, 0.081) |
| Interdaily Stability | 0.027 (0.007, 0.048) | 0.023 (0.006, 0.04) | -0.004 (-0.026, 0.018) |
| Intradaily Variability | -0.025 (-0.061, 0.011) | -0.028 (-0.059, 0.002) | -0.003 (-0.041, 0.035) |
| Self-Report Sleep Midpoint | -0.088 (-0.295, 0.119) | -0.151 (-0.332, 0.031) | -0.062 (-0.301, 0.176) |
| Actigraphy Midpoint | -0.058 (-0.279, 0.164) | -0.069 (-0.247, 0.109) | -0.011 (-0.249, 0.227) |
| Acrophase | 0.101 (-0.137, 0.339) | 0.143 (-0.05, 0.337) | 0.042 (-0.209, 0.293) |
| **Age 91**^b^ | | | |
| **Feature** | **MCI vs. Normal** | **Dementia vs. Normal** | **Dementia vs. MCI** |
| Self-Report Total Sleep Time | 0.322 (-0.041, 0.685) | **0.660 (0.214, 1.105)** | 0.338 (-0.169, 0.845) |
| Actigraphy Rest Interval Length | 0.03 (-0.096, 0.156) | -0.147 (-0.334, 0.04) | -0.177 (-0.38, 0.027) |
| Alpha | -0.058 (-0.132, 0.016) | -0.004 (-0.086, 0.077) | 0.054 (-0.045, 0.152) |
| Actigraphy Midpoint SD | 0.049 (-0.078, 0.175) | -0.09 (-0.228, 0.047) | -0.139 (-0.303, 0.025) |
| Interdaily Stability | 0.009 (-0.022, 0.04) | 0.004 (-0.028, 0.036) | -0.005 (-0.043, 0.033) |
| Intradaily Variability | 0.015 (-0.034, 0.063) | 0.022 (-0.028, 0.072) | 0.007 (-0.054, 0.069) |
| Self-Report Sleep Midpoint | -0.033 (-0.278, 0.212) | -0.154 (-0.434, 0.127) | -0.12 (-0.446, 0.206) |
| Actigraphy Midpoint | 0.038 (-0.274, 0.349) | 0.165 (-0.167, 0.497) | 0.127 (-0.262, 0.517) |
| Acrophase | -0.012 (-0.355, 0.331) | -0.068 (-0.431, 0.296) | -0.056 (-0.493, 0.382) |

^a^Assumes that all pathways have normal cognition. Estimates are adjusted for average levels of all covariates.

^b^Assumes that participants on the Dementia pathway have dementia, participants on the Stable MCI pathway have MCI, and participants on the Normal pathway have no cognitive diagnosis. Estimates are adjusted for average levels of all covariates.

*Note: Note: Bold figures are statistically significant.*

# **Table S9a.** Sensitivity analysis 4: Estimated within-pathway changes and 95% Confidence Intervals (*d* [95% CI]) in self-report sleep, RAR, and actigraphy sleep features. Estimates assume a starting age of 80 and end age of 94.

| **Feature** | **Normal Path**^a^ | **Stable MCI Path**^b^ | **Dementia Path**^c^ |
| --- | --- | --- | --- |
| *Amount* | | | |
| Self-reported Total Sleep Time | -0.11 (-0.282, 0.062) | 0.321 (-0.165, 0.806) | **1.243 (0.67, 1.817)** |
| Actigraphy Rest Interval Length | 0.018 (-0.057, 0.094) | **0.266 (0.041, 0.491)** | 0.234 (-0.084, 0.552) |
| Alpha | **0.085 (0.055, 0.115)** | 0.06 (-0.038, 0.158) | **0.144 (0.042, 0.247)** |
| *Regularity* | | | |
| Actigraphy Midpoint St.Dev | **0.148 (0.066, 0.23)** | **0.232 (0.046, 0.418)** | 0.06 (-0.117, 0.237) |
| Interdaily Stability | **-0.027 (-0.05, -0.003)** | -0.043 (-0.088, 0.002) | **-0.059 (-0.098, -0.021)** |
| Intradaily Variability | **0.218 (0.191, 0.244)** | **0.289 (0.218, 0.36)** | **0.327 (0.255, 0.4)** |
| *Timing* | | | |
| Self-reported Midpoint | 0.006 (-0.157, 0.168) | 0.096 (-0.231, 0.423) | -0.053 (-0.428, 0.321) |
| Actigraphy Rest Interval Midpoint | **-0.830 (-1.037, -0.623)** | **-0.705 (-1.106, -0.305)** | **-0.485 (-0.885, -0.085)** |
| Acrophase | **-0.471 (-0.621, -0.321)** | **-0.622 (-1.04, -0.203)** | **-0.752 (-1.19, -0.314)** |

**^a^**Estimates are for a ‘typical’ participant on the Normal pathway, with no cognitive diagnosis across follow-up and average levels of covariates.

**^b^**Estimates are for a ‘typical’ participant on the Stable MCI path, with MCI at age 86 and no dementia diagnosis.

**^c^**Estimates are for a ‘typical’ participant on the Dementia path, with MCI at age 86 and a dementia diagnosis at age 90.

*Note: Note: Bold figures are statistically significant.*

# **Table S9b.** Sensitivity analysis 4: Standardized between-pathway differences at benchmark ages 80 and 94.

| **Age 80**^a^ | | | |
| --- | --- | --- | --- |
| **Feature** | **Normal vs. MCI** | **Normal vs. Dementia** | **MCI vs. Dementia** |
| Self-Report Total Sleep Time | -0.014 (-0.284, 0.255) | **-0.382 (-0.618, -0.146)** | **-0.368 (-0.687, -0.049)** |
| Actigraphy Rest Interval Length | -0.074 (-0.175, 0.026) | **-0.139 (-0.225, -0.052)** | -0.064 (-0.171, 0.043) |
| Alpha | -0.04 (-0.085, 0.006) | **-0.049 (-0.086, -0.012)** | -0.009 (-0.057, 0.039) |
| Actigraphy Midpoint SD | -0.011 (-0.096, 0.073) | -0.024 (-0.091, 0.042) | -0.013 (-0.105, 0.079) |
| Interdaily Stability | **0.036 (0.014, 0.058)** | **0.024 (0.007, 0.042)** | -0.012 (-0.035, 0.012) |
| Intradaily Variability | -0.025 (-0.061, 0.011) | -0.028 (-0.059, 0.002) | -0.003 (-0.041, 0.035) |
| Self-Report Sleep Midpoint | -0.054 (-0.282, 0.174) | -0.161 (-0.36, 0.038) | -0.107 (-0.376, 0.162) |
| Actigraphy Midpoint | -0.055 (-0.297, 0.187) | -0.102 (-0.292, 0.088) | -0.046 (-0.31, 0.217) |
| Acrophase | 0.101 (-0.137, 0.339) | 0.143 (-0.05, 0.337) | 0.042 (-0.209, 0.293) |
| **Age 94**^b^ | | | |
| **Feature** | **Normal vs. MCI** | **Normal vs. Dementia** | **MCI vs. Dementia** |
| Self-Report Total Sleep Time | 0.417 (-0.041, 0.874) | **0.971 (0.399, 1.543)** | 0.555 (-0.105, 1.214) |
| Actigraphy Rest Interval Length | 0.173 (-0.053, 0.398) | 0.077 (-0.242, 0.396) | -0.095 (-0.469, 0.278) |
| Alpha | -0.064 (-0.159, 0.030) | 0.011 (-0.094, 0.115) | 0.075 (-0.055, 0.204) |
| Actigraphy Midpoint SD | 0.073 (-0.094, 0.240) | -0.112 (-0.292, 0.067) | -0.185 (-0.405, 0.034) |
| Interdaily Stability | 0.020 (-0.024, 0.064) | -0.008 (-0.05, 0.033) | -0.028 (-0.08, 0.023) |
| Intradaily Variability | 0.046 (-0.027, 0.119) | **0.081 (0.006, 0.156)** | 0.035 (-0.062, 0.133) |
| Self-Report Sleep Midpoint | 0.036 (-0.283, 0.356) | -0.22 (-0.592, 0.151) | -0.256 (-0.687, 0.174) |
| Actigraphy Midpoint | 0.069 (-0.319, 0.458) | 0.243 (-0.178, 0.664) | 0.174 (-0.326, 0.674) |
| Acrophase | -0.049 (-0.474, 0.376) | -0.138 (-0.595, 0.319) | -0.088 (-0.651, 0.474) |

^a^Assumes that all pathways have normal cognition. Estimates are adjusted for average levels of all covariates.

^b^Assumes that participants on the Dementia pathway have dementia, participants on the Stable MCI pathway have MCI, and participants on the Normal pathway have no cognitive diagnosis. Estimates are adjusted for average levels of all covariates.

*Note: Note: Bold figures are statistically significant.*
